# Supplementary material for: Creeping Stem 1 regulates directional auxin transport for lodging resistance in soybean
Source: Plant Biotechnol J. 2024 Nov 13;23(2):377–94. doi: 10.1111/pbi.14503 (PMC11772330; doi:10.1111/pbi.14503)
Supplement: Supplementary file 1 — Figure S1 Field phenotype of the cs1 mutant. The red arrow points to the specific lodging position. Figure S2 Diversity of agronomic traits between NN1138‐2 and cs1. Comparison of main stem length (a), number of main stem nodes (b), number of pods per plant (c), seeds per plant (d), grain weight per plant (e) and hundred‐grain weight (f) between NN1138‐2 and cs1. The asterisks denote statistically significant differences from the wild type by a two‐sided t‐test (**P < 0.01). Values are means ± SD (n > 18). Figure S3 The gravity response capability of the wild‐type NN1138‐2 and cs1 mutant. (a) Hypocotyl Gravitropic response overtime in darkness. Seven‐day‐old seedlings were placed horizontally in darkness and the gravitropic response of hypocotyl was recorded at intervals of 1 h, 4 h and 15 h. (b) Gravitropic phenotypes of the cs1 mutant and wild‐type NN1138‐2 under light conditions. Values are means ± SD (n = 3). The asterisks indicate a statistically significant difference from the wild type as determined by a two‐sided t‐test (*P < 0.05). Figure S4 Hypocotyl gravitropism patterns. (a) Hypocotyl gravitropism pattern diagram. I: Plants grown for 3.5 days were marked at intervals of 0.5 cm from the growth point. II: The distances between each mark were measured after 24 h, and the hypocotyls were sectioned into 0.5‐cm increments from the growth point. III: The sectioned plants were placed horizontally in darkness. After 10 h, the bending at the marked portions was observed. The bend is indicated in grey. (b) Bending photographs of the sectioned hypocotyls of indicated lines. Figure S5 Identification of the CS1 gene in soybeans. (a) Verification of mutation sites in isolated populations. The isolated population of lodging plants showed consistency with the parental mutant mutation sites. Red indicates the SNP site, with base C representing the wild type and T representing the mutant plants respectively. (b) CS1 protein sequence length pattern in wild type. An orange b [file PBI-23-377-s001.docx]

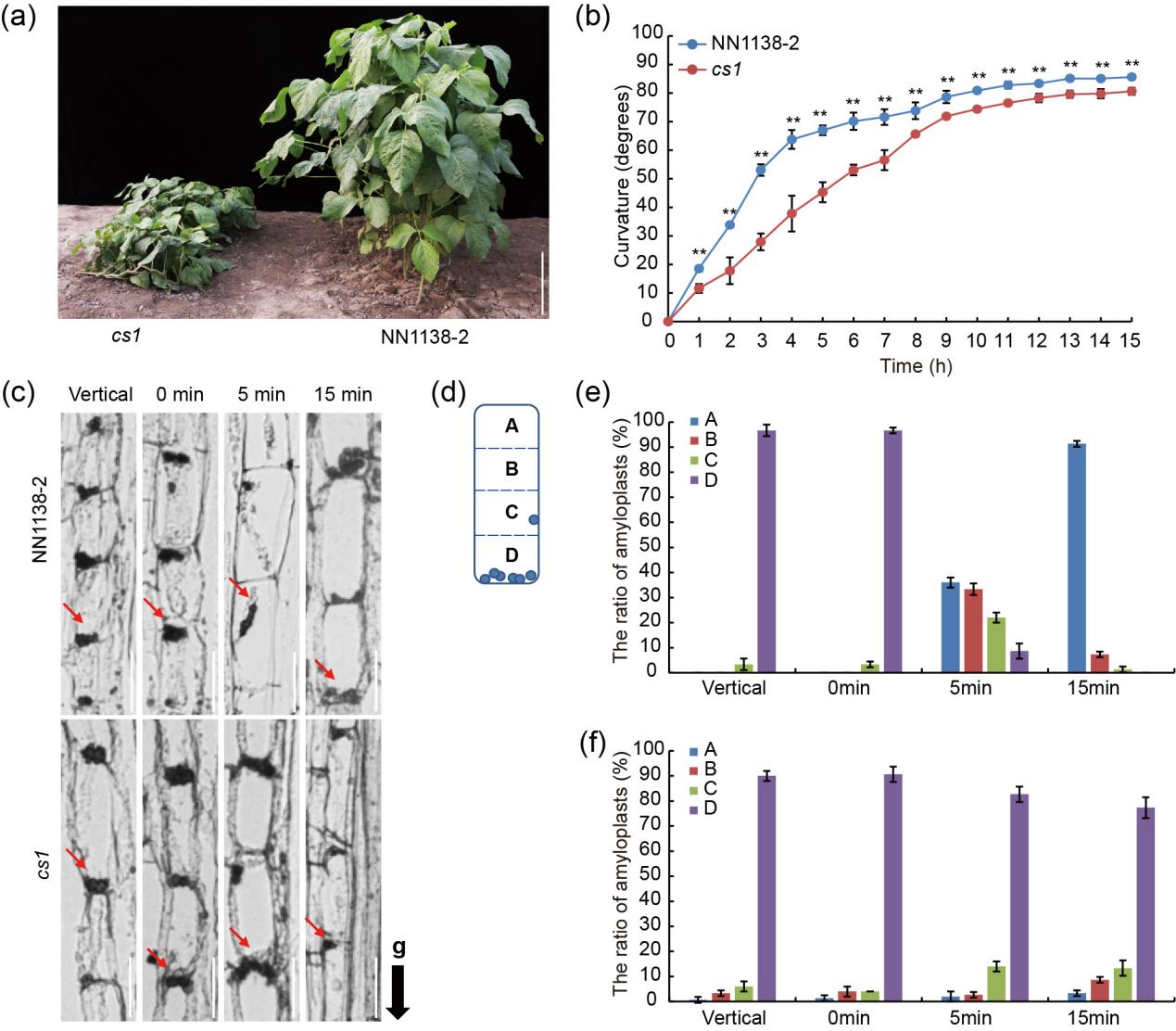
Figure 1. Identification of the *cs1* mutant. (a) 7-week-old plants of the *cs1* mutant and the wild-type accession NN1138-2 at vegetative stage in the field. Scale bars = 20 cm. (b) Gravitropic response of hypocotyl in dark. The indicated lines were grown under LD conditions for 7 days. Values are means ± s.d. (*n* = 3). The significant difference between the wild type and *cs1* mutant at each time point was determined by a two-sided *t*-test (** *P* < 0.01). (c) Longitudinal section images show the distribution of amyloplasts in endodermal cells. The 7-day-old vertical growing plants were inverted for 0 to 15 min. The fragments of hypocotyls (1-2 cm below the cotyledon node) were collected and fixed with the gravity direction maintained at the indicated time point. The red arrows indicate the locations of the amyloplasts, and the black arrow indicates the direction of gravity (g). Scale bars = 5 µm. (d) Schematic diagram of an endodermal cell, partitioned into four blocks (A, B, C and D from top to bottom) for quantitative analysis, blue circles represent amyloplasts. (e-f) The ratio of amyloplasts in each block in NN1138-2 (e) and *cs1* (f), Data are means ± s.d. (*n* = 50).



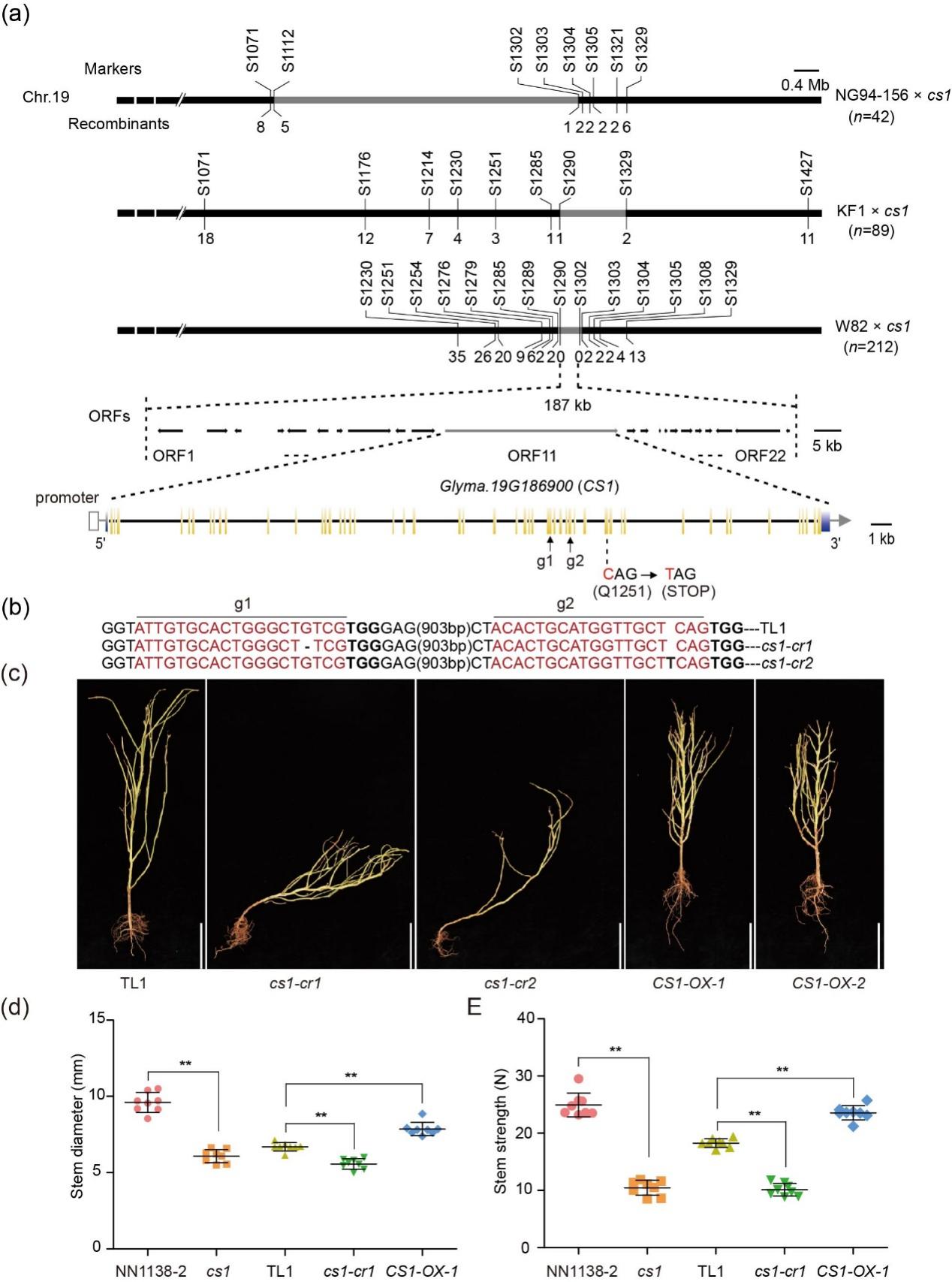


Figure 2. *CS1* encodes a MAESTRO-RELATED HEAT DOMAIN-CONTAINING protein. (a) Fine mapping of *CS1* candidate gene using three populations: NG94-156 × *cs1*, KF1 × *cs1*, and W82 × *cs1*. The *CS1* locus was narrowed down to a 187-kb region containing 22 annotated ORFs (open reading frame). A nonsense mutation (C to T) was identified in the 39th exon of *Glyma.19G186900* in the *cs1* mutant*,* leading to a premature protein at Q1252. The *Glyma.19G186900* gene structure is schematically shown; exons and introns are indicated by yellow bars and black bars respectively. The blue bars indicated the 5´- and 3´-untranslated region. (b) Two sgRNAs (g1 and g2, black arrows) were designed to target the 32th and 36th exons of *Glyma.19G186900* respectively. The mutant sequences of two representative homozygous mutants (*cs1-cr1* and *cs1-cr2*) at T_2_ generation are shown. The target sites of sgRNA are highlighted in red letters with the protospacer-adjacent motif (PAM) in bold. The black base and dash line within the target sites denote nucleotide insertion and deletion respectively. (c) Plant architectures of the wild type (TL1), *CS1* knockout mutants (*cs1-cr1*, *cs1-cr2*), and *CS1* overexpression lines (*CS1-OX-1* and *CS1-OX-2*) at mature stage in the field. Scale bar = 25 cm. (d-e) Comparison of hypocotyl diameter (d) and stem length (e) of indicated lines at 46 days after sowing. Data are means ± s.d. (*n* = 8). The significant difference between the indicated line and wild type was determined by two-sided *t*-test (* *P* < 0.05; ** *P* < 0.01).


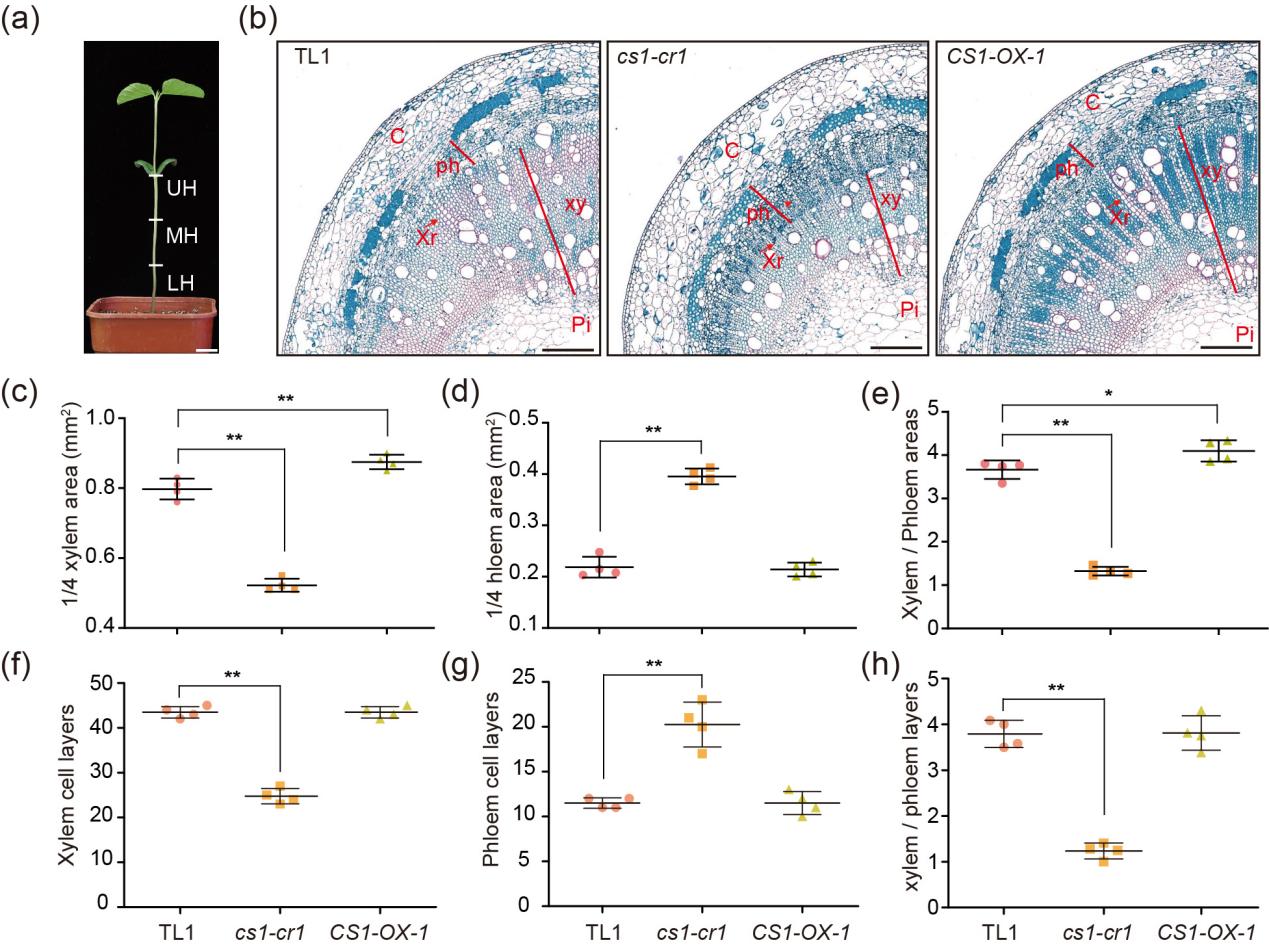


Figure 3. The *CS1* gene regulates xylem and phloem development in hypocotyl. (a) Pattern of hypocotyl Segmentation. (b) Quadrant cross section images show the cell layer structures in the upper region of hypocotyls. The plants of indicated lines were grown under LD conditions for 7 days. C, cortex; ph, phloem; xr, xylem rays; xy, xylem; pi, pith. The red arrows represent xylem rays. Scale bar = 250 µm. (c-h) Scatter plots of xylem areas (c), phloem areas (d), xylem areas / phloem areas (e), xylem cell lays (f), phloem cell lays (g), and xylem cell lays / phloem cell lays (h) of indicated lines as in (b). Data are means ± s.d. (*n* = 4). The significant difference between the indicated line and wild type was determined by two-sided *t*-test (**P* < 0.05; ***P* < 0.01).


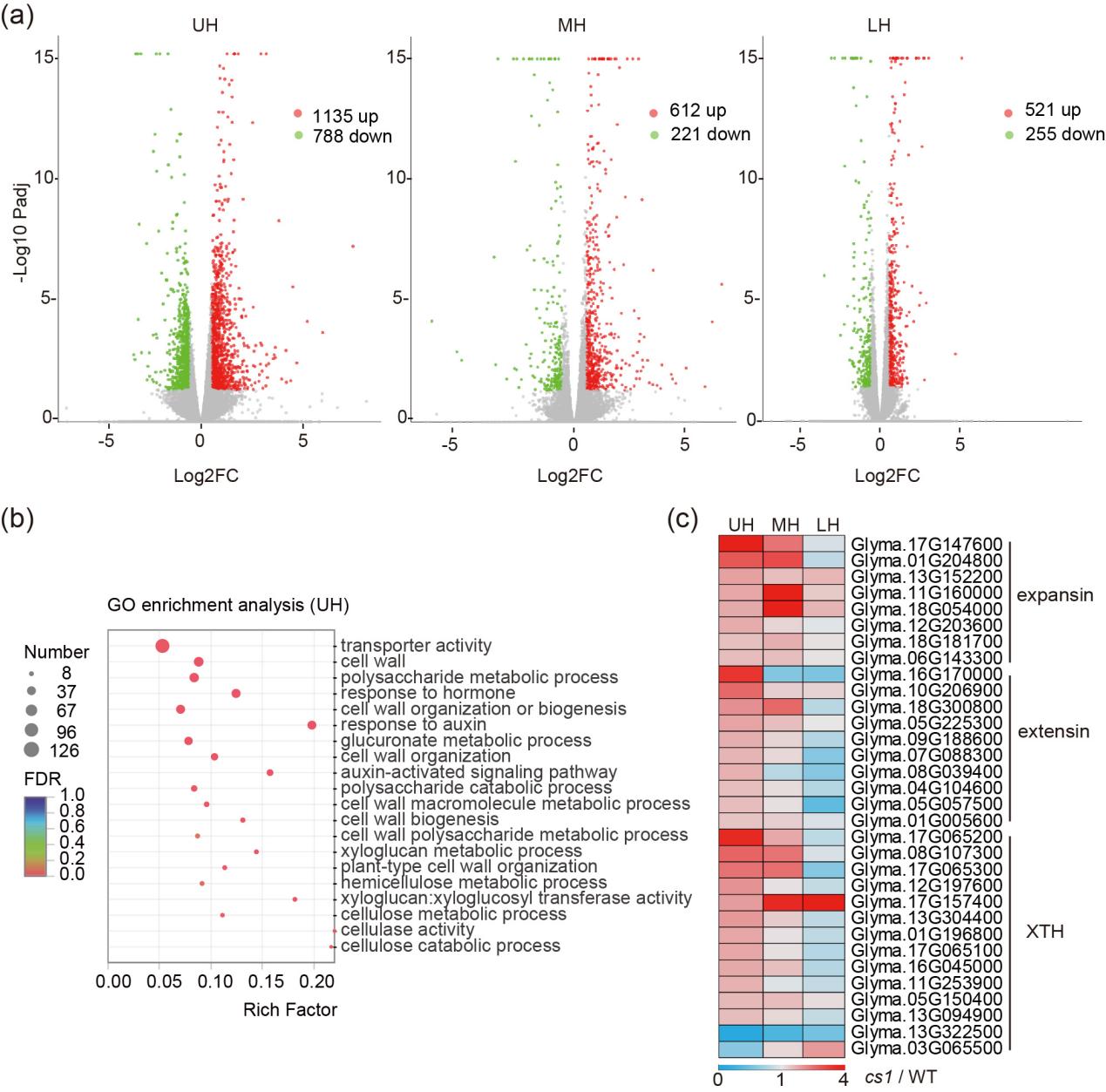


Figure 4. The *CS1* gene affects cell wall related genes and auxin transport related genes changes in the upper hypocotyl. (a) Volcano plot of gene expression differences between the NIL lines in the indicated segments of hypocotyls. The abscissa is the multiple of the difference of gene / transcript expression between the two samples, and the ordinate is the statistical test value of the difference of gene expression, namely p value. Each dot in the plot represents a specific gene, the red dot represents the significantly up-regulated gene, the blue dot represents the significantly down-regulated gene, and the gray dot represents the non-significant differential gene. (b) GO term related to cell wall and auxin in UH. The GO terms related to cell wall and auxin in UH were plotted by GO IDs. The vertical axis represents the GO term, horizontal axis represents the Rich factor [the ratio of the number of genes / transcripts enriched in the GO term (Sample number) to the number of annotated genes / transcripts (Background number)]. The larger the Rich factor, the greater the degree of enrichment. The size of the dot indicates the number of genes / transcripts in the GO Term, and the color of the dot corresponds to different Padjust (*P* value-corrected) ranges. C) Heat map of expansin, extensin and XTH gene families (involved in cell wall organization and modiﬁcation) in UH, MH and LH. Plotted with the TPM*^cs1^*/TPM^WT^ of the corresponding part, red represents up-regulation > 1, light blue represents down-regulation < 1, and gray represents no change = 1.


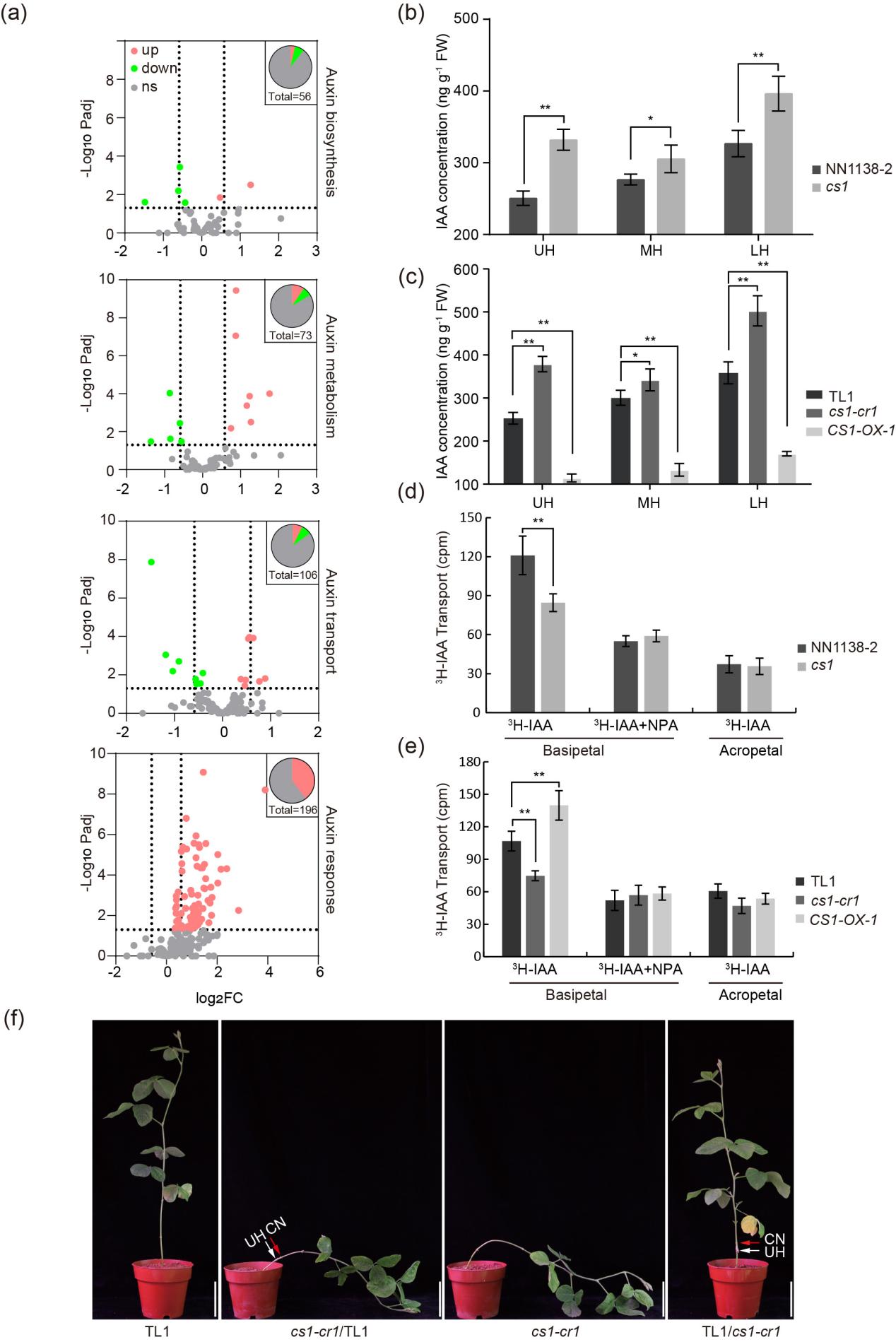


Figure 5. The slow PAT in *cs1* results in the increase of auxin concentration and gradient disorder. (a) Volcanic map of auxin biosynthesis, metabolism, transport and response related genes in UH. The abscissa represents the fold change of gene expression difference between the two samples, while the ordinate represents the statistical test value (p-value) of the difference in gene expression change. Each point in the figure represents a specific gene, where red dots indicate significantly up-regulated genes, green dots indicate significantly down-regulated genes, and gray dots represent non-significantly different genes. The two dashed lines on the abscissa represent 1.5 times up- and downregulation of genes, respectively, while the dashed line on the ordinate represents p = 0.05. (b-c) Auxin concentration in each segment of hypocotyls of indicated lines. FW means fresh weight. Values are means ± s.d. (*n* ≥ 5). (d-e) Comparison of PAT differences in hypocotyls among indicated lines. Values are means ± s.d. (*n* =5). Comparison of differences in PAT among TL1, *cs1-cr1*, and *CS1-OX-1*. Values are means ± s.d. (*n* =5). B-E, The asterisks indicate statistically significant difference from the wild type by two-sided *t*-test (* *P* <0.05; ** *P* <0.01). (f) Grafting leads to *cs1-cr1* not lodging, but TL1 lodging. The white arrow indicates the grafting position, and the red arrow indicates the cotyledon node. CN means cotyledon node. Representative images of at least three independent grafting experiments of indicated combination. *cs1-cr1*/TL1: *cs1-cr1* is scion, TL1 is rootstock; TL1/ *cs1-cr1*:TL1 is scion, *cs1-cr1* is rootstock. Scale bar = 10 cm.


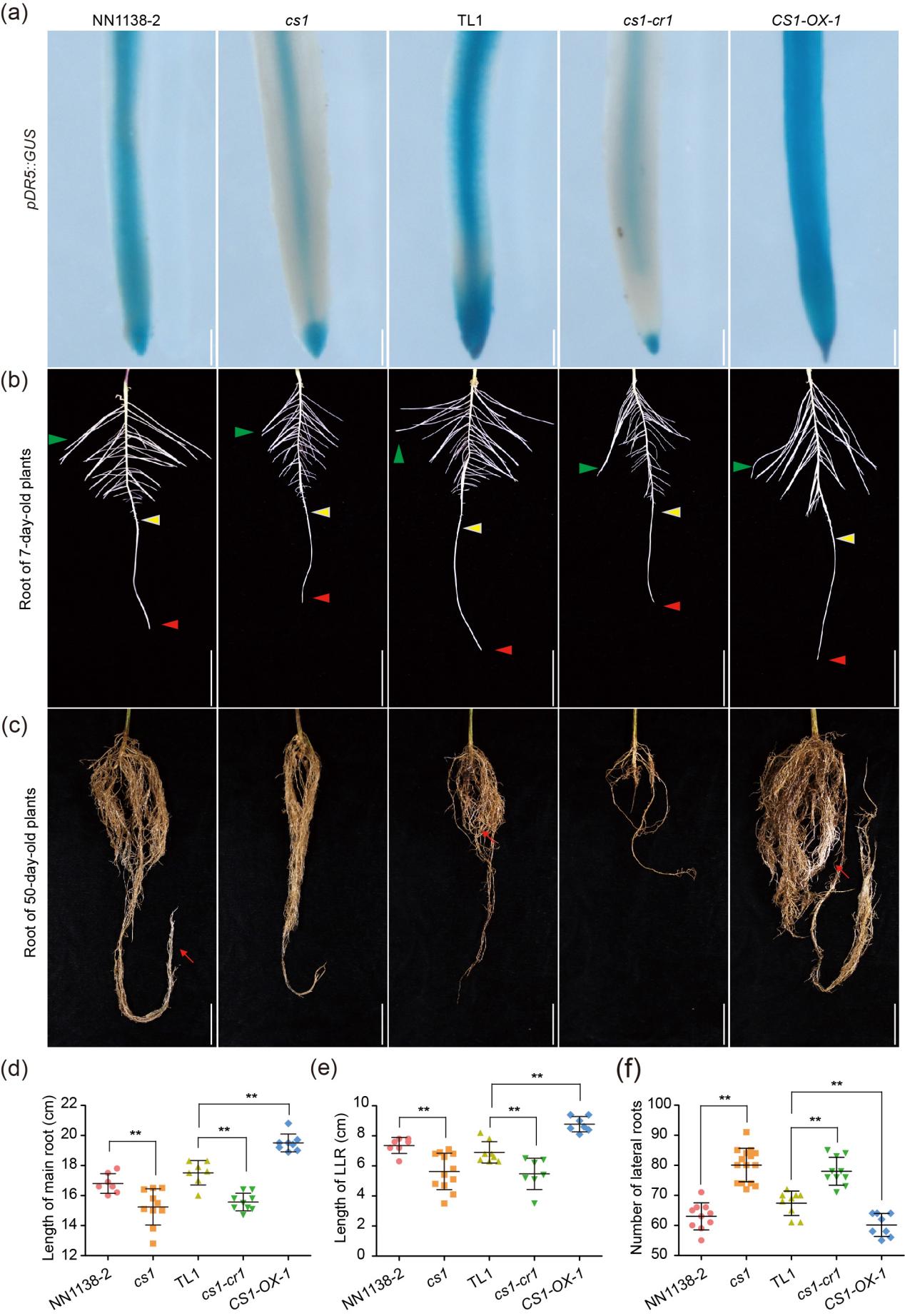


Figure 6. Dysfunction of *CS1* confers abnormal auxin distribution and root morphology.

(a) Histochemical staining of *pDR5::GUS* transgenic hairy roots in the indicated backgrounds. Three independent experiments yielded similar results. Scale bar = 50 µm. (b) Root images of 7-day-old seedlings grown under LD conditions. The red triangle represents the tip of the main root, the green triangle represents the longest lateral root, and the yellow triangle represents the newest lateral root. Scale bar = 4 cm. (c) Representative root images of indicated genotypes grown under LD conditions for 50 days. Scale bar = 3 cm. (d-f) The main root length, the longest lateral root length (data from b), and the number of lateral roots (data from b). Values are means ± s.d. (*n* = 10). The asterisks indicate statistically significant difference from the wild type by two-sided *t*-test (** *P* <0.01).


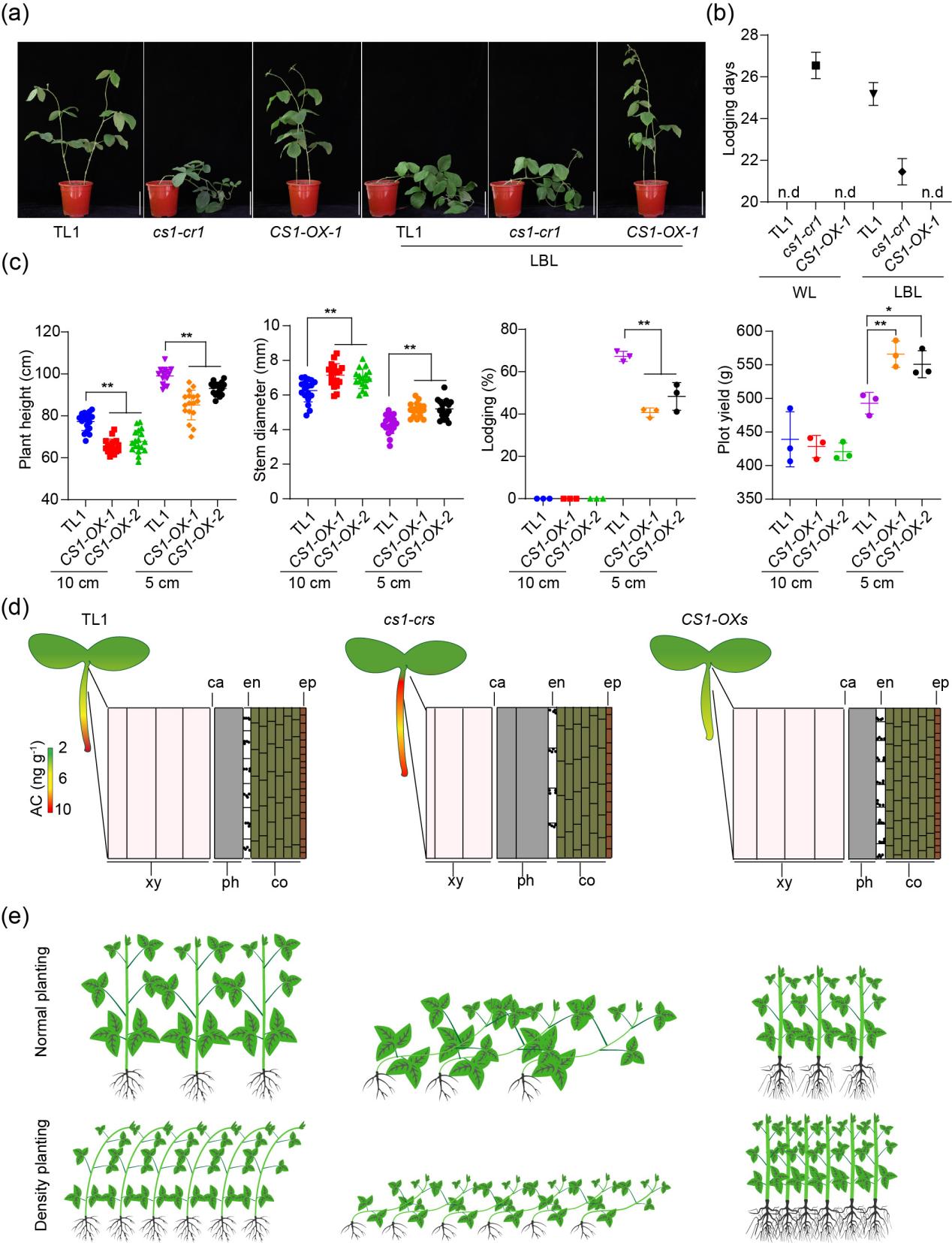


Figure 7. Density planting exacerbated TL1 lodging, but had little effect on *CS1-OXs*.

(a) Lodging phenotype under wight light (WL) and low blue light (LBL). Scale bar = 9 cm. (b) Plant lodging days in a. Values are means ± s.d. (*n* = 4). (c) Comparison of the plant height, stem diameter, lodging rate and plot yield of TL1 and *CS1-OXs* under normal planting and density planting. Normal planting means 10 cm space. Density planting means 5 cm space. Plants at maturity stage R8 whose main stem leaned more than 45° were recorded as having lodged. Data are means ± s.d of three biological repeats. The significant difference between the indicated line and wild type was determined by two-sided *t*-test (* *P* < 0.05, ** *P* < 0.01). (d) Auxin concentration gradient and organization structure pattern diagram in TL1, *cs1-cr1* and *CS1-OX-1*. The black circles in en represent amyloplasts, and the position represents the distribution of amyloplasts after plants were inverted 5 minutes. Xy, xylem; ca, the cambium; ph, phloem; en, endodermis; co, cortex; ep, epidermis. AC means auxin concentration. (e) The morphology of TL1, *cs1-cr1* and *CS1-OX-1* at different planting densities.


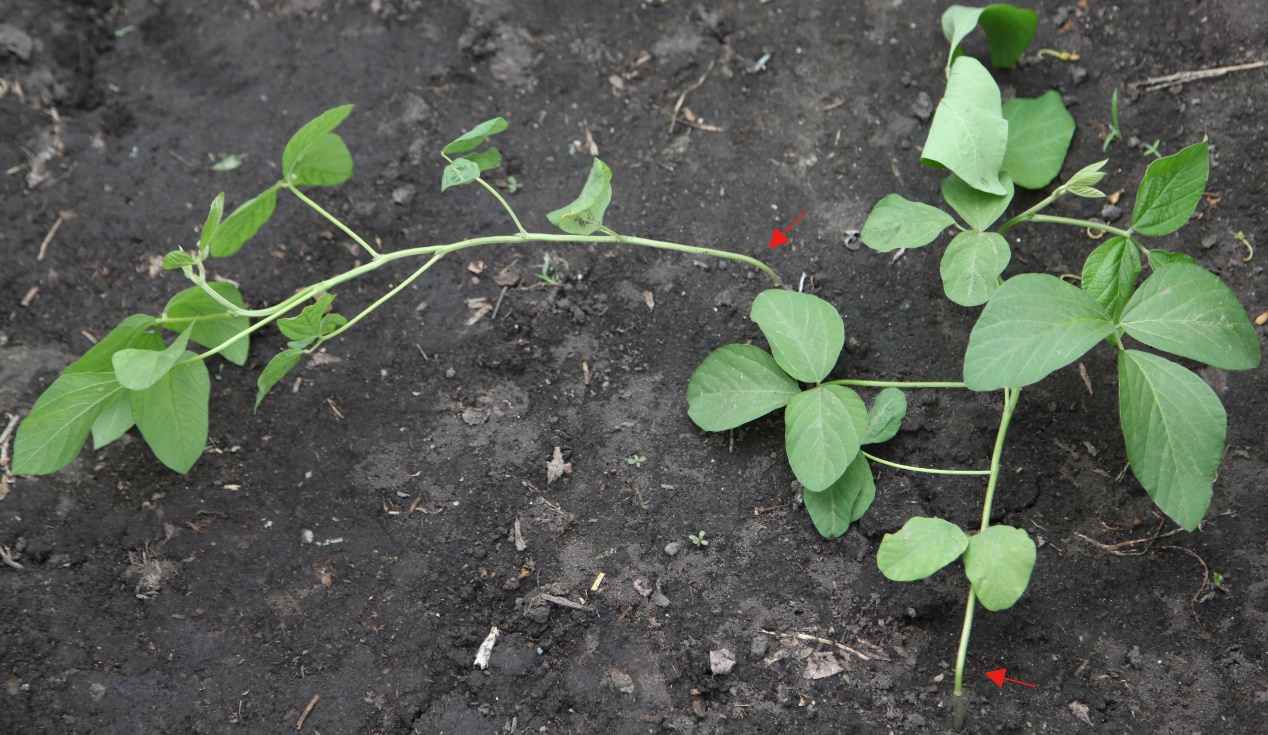


Figure S1. Field phenotype of the *cs1* mutant. The red arrow points to the specific lodging position.


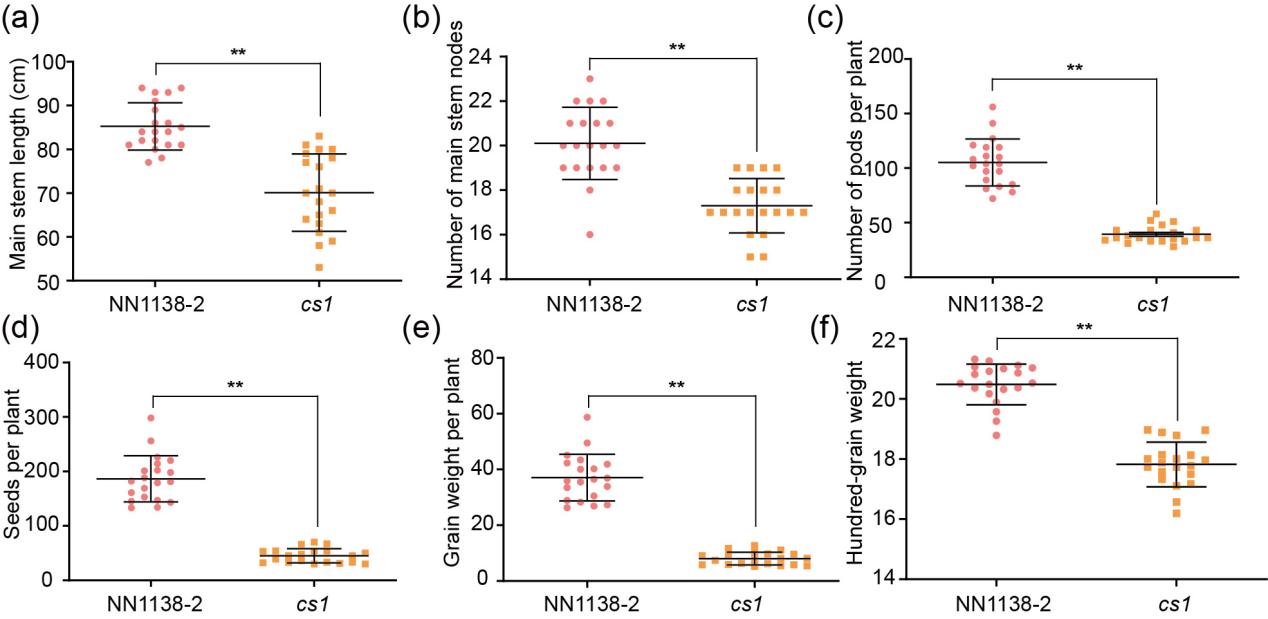


Figure S2. Diversity of agronomic traits between NN1138-2 and *cs1*. Comparison of main stem length (a), number of main stem nodes (b), number of pods per plant (c), seeds per plant (d), grain weight per plant (e) and hundred-grain weight (f) between NN1138-2 and *cs1*. The asterisks denote statistically significant differences from the wild type by a two-sided *t*-test (** *P* < 0.01). Values are means ± s.d. (*n* > 18).


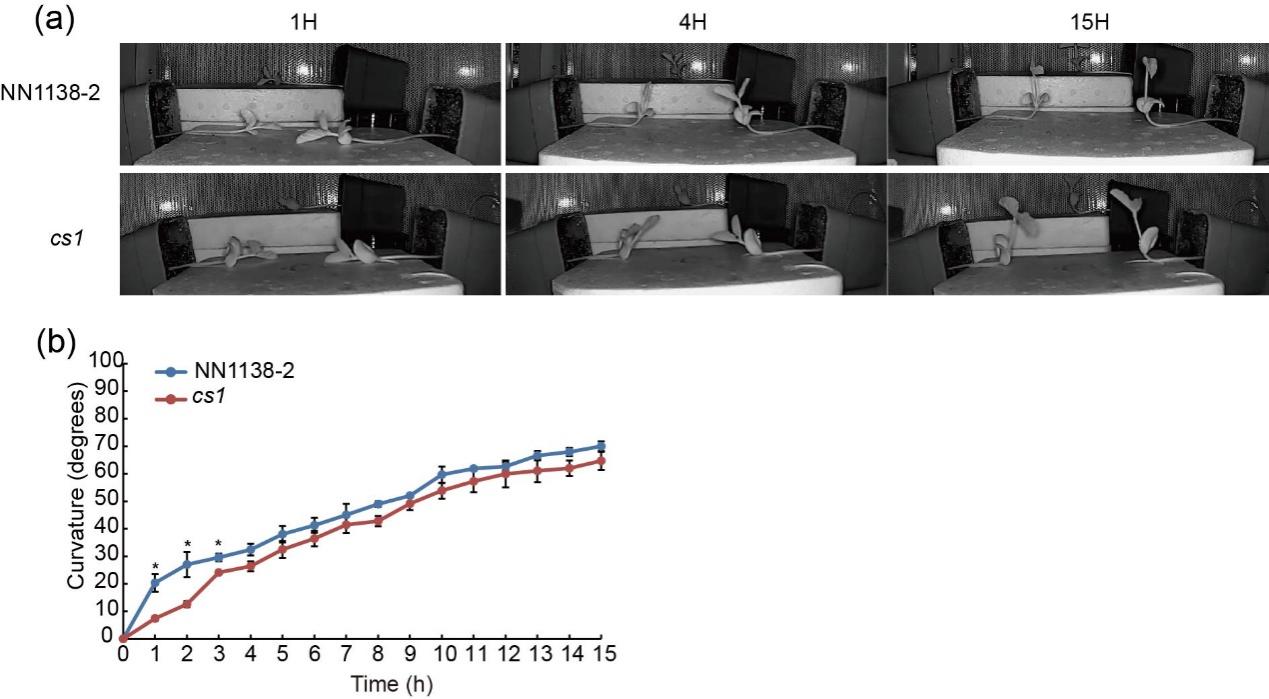


Figure S3. The gravity response capability of the wild-type NN1138-2 and *cs1* mutant. (a) Hypocotyl Gravitropic response overtime in darkness. Seven-day-old seedlings were placed horizontally in darkness and the gravitropic response of hypocotyl were recorded at intervals of 1 hour, 4 hours and 15 hours. (b) Gravitropic phenotypes of the *cs1* mutant and wild type NN1138-2 under light condition. Values are means ± SD (*n* = 3). The asterisks indicate statistically significant difference from the wild type as determined by a two-sided *t*-test (* *P* < 0.05).


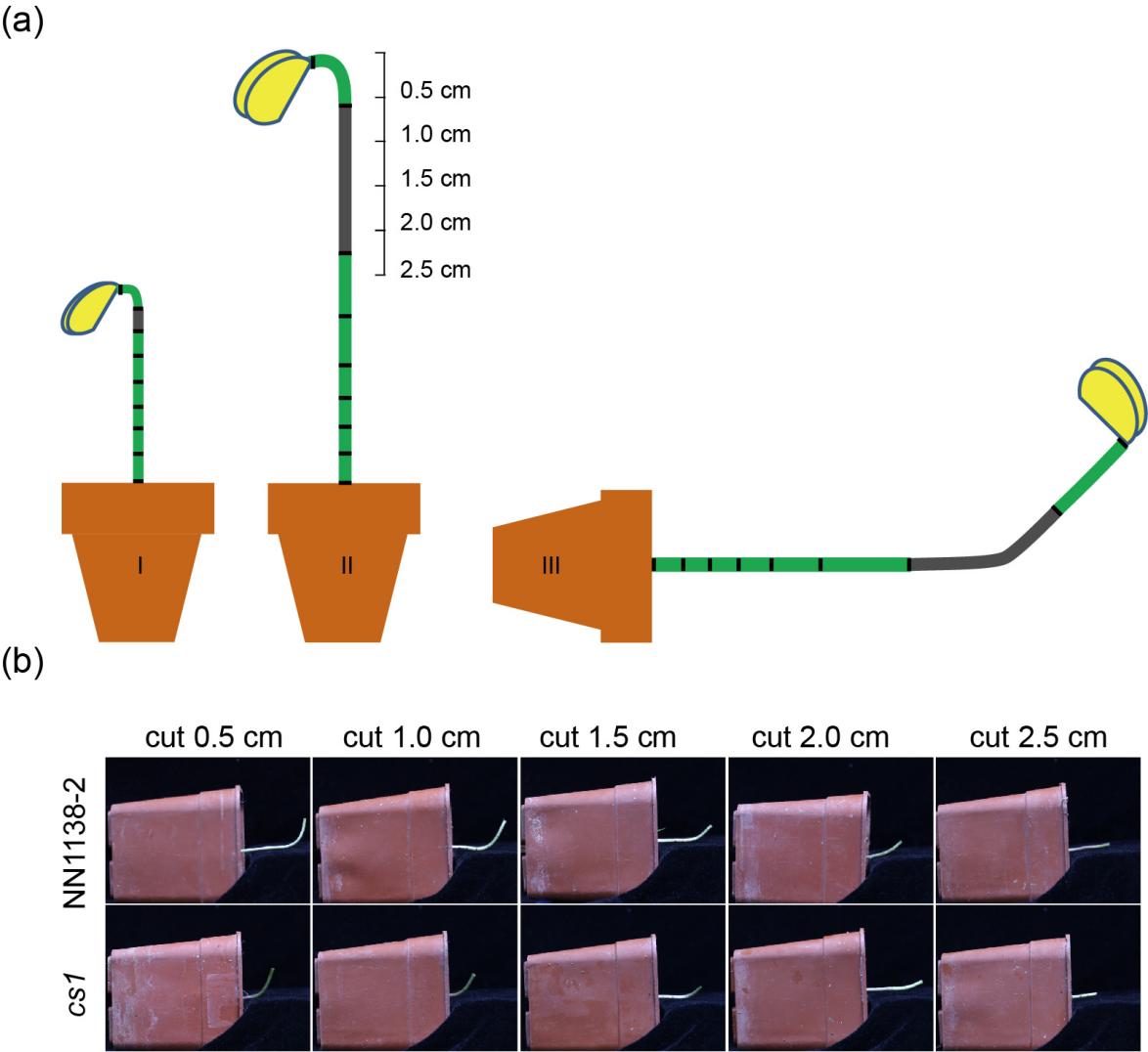


Figure S4. Hypocotyl gravitropism patterns. (a) Hypocotyl gravitropism pattern diagram. I: Plants grown for 3.5 days were marked at intervals of 0.5 cm from the growth point. II: The distances between each mark were measured after 24 hours, and the hypocotyls were sectioned into 0.5 cm increments from the growth point. III: The sectioned plants were placed horizontally in darkness. After 10 hours, the bending at the marked portions was observed. The bend is indicated in gray. (b) Bending photographs of the sectioned hypocotyls of indicated lines.


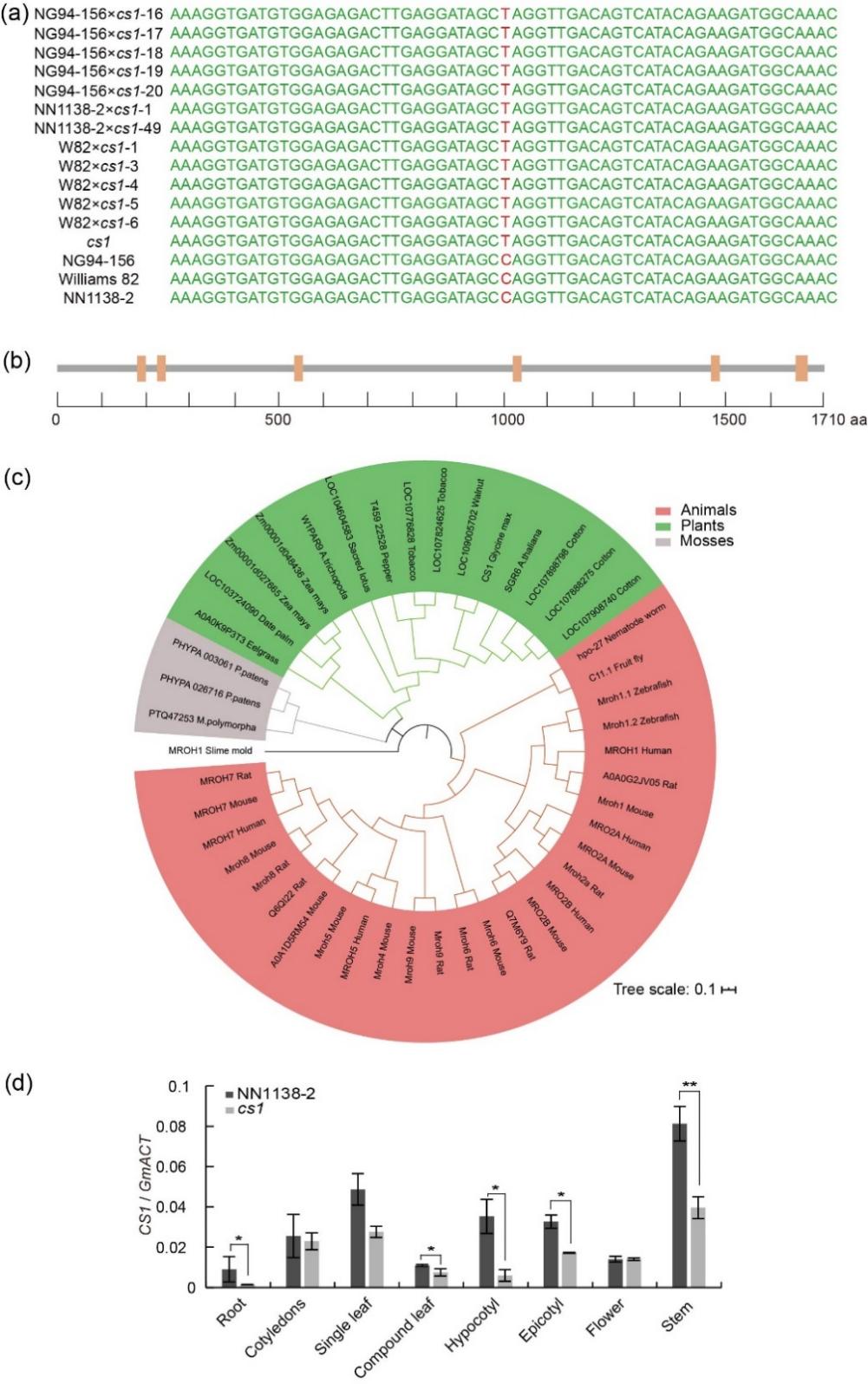


Figure S5. Identification of the *CS1* gene in soybeans. (a) Verification of mutation sites in isolated populations. The isolated population of lodging plants showed consistency with the parental mutant mutation sites. Red indicates the SNP site, with base C representing the wild type and T representing the mutant plants respectively. (b) CS1 protein sequence length pattern in wild type. An orange box represents a HEAT repeat within the protein sequencing. (c) Phylogenetic tree of CS1 across animals, plants, and mosses. (d) Tissue-specific expression patterns of *CS1* in NN1138-2 and *cs1*. *GmActin11* was used as a control in the real-time PCR analyses. The asterisks indicate statistically significant difference from the wild type by two-sided *t*-test (* *P* < 0.05; ** *P* < 0.01).


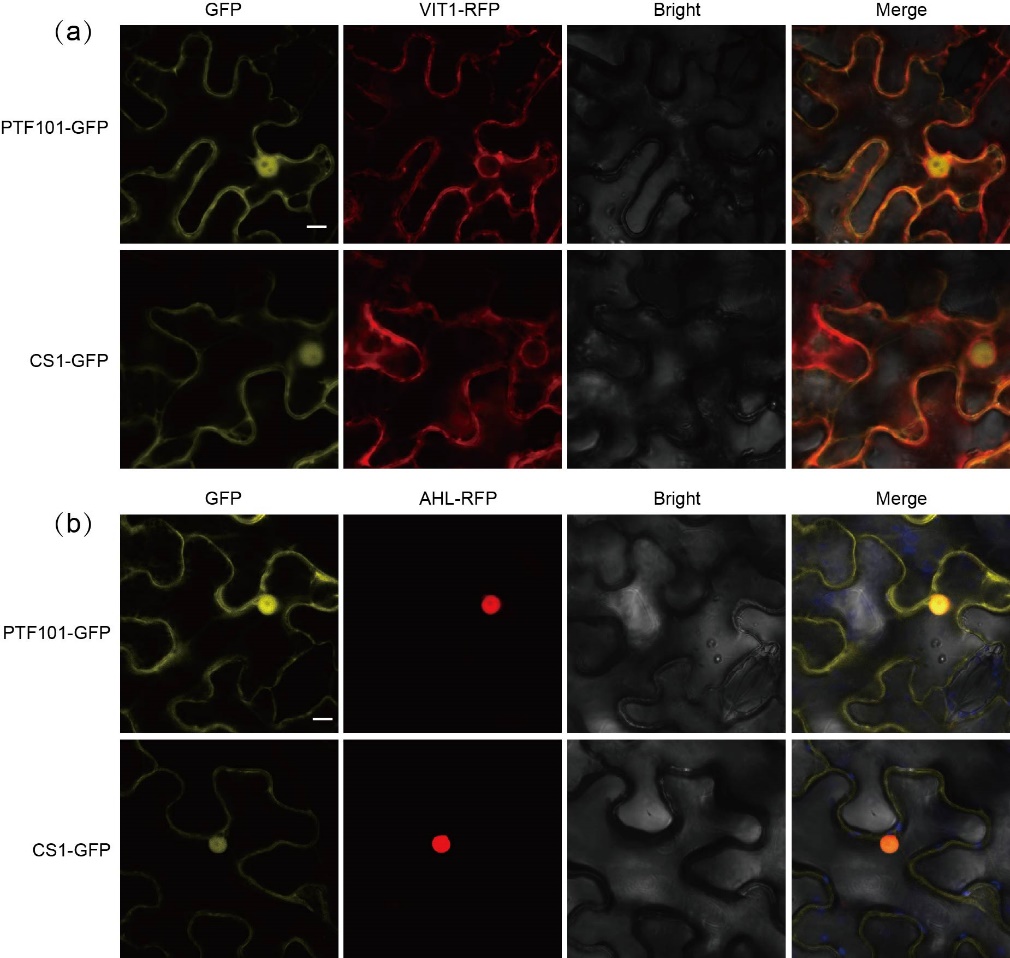


Figure S6. Subcellular localization pattern of the CS1 proteins in tobacco leaf epidermal cells. (a-b) Soybean CS1 protein is localized to the vacuolar membrane (a) and to the nuclear (b). GFP proteins expressed by the PTF101-GFP vector serves as a control for protein expression and localization. The Arabidopsis thaliana VIT1 protein fused with RFP serves as vacuole-specific marker, while the AHL protein fused with RFP is used as a nuclear marker. Scale bars, 10 μm.


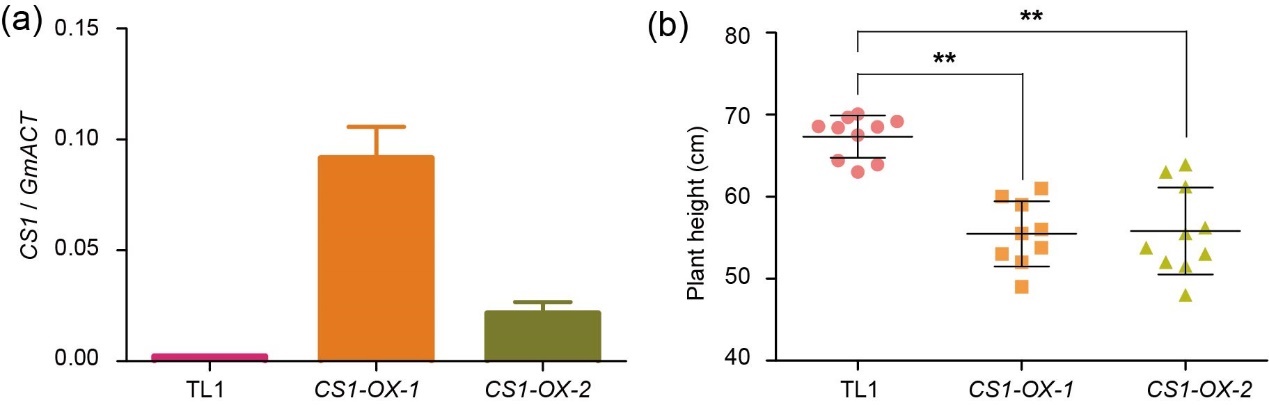
Figure S7. Plant height phenotype of *CS1* over-expression lines. (a) Comparison of *CS1* expression levels between the wild type (TL1) and transgenic materials (*CS1-OX-1*, *CS1-OX-2*). *GmActin11* was used as a control gene in the real-time PCR analyses. Values are means ± s.d. (*n* = 3). (b) Plant height comparison between the indicated lines. Values are means ± s.d. (*n* > 9). The asterisks indicate statistically significant differences from the wild type by two-sided *t*-test (** *P* < 0.01).


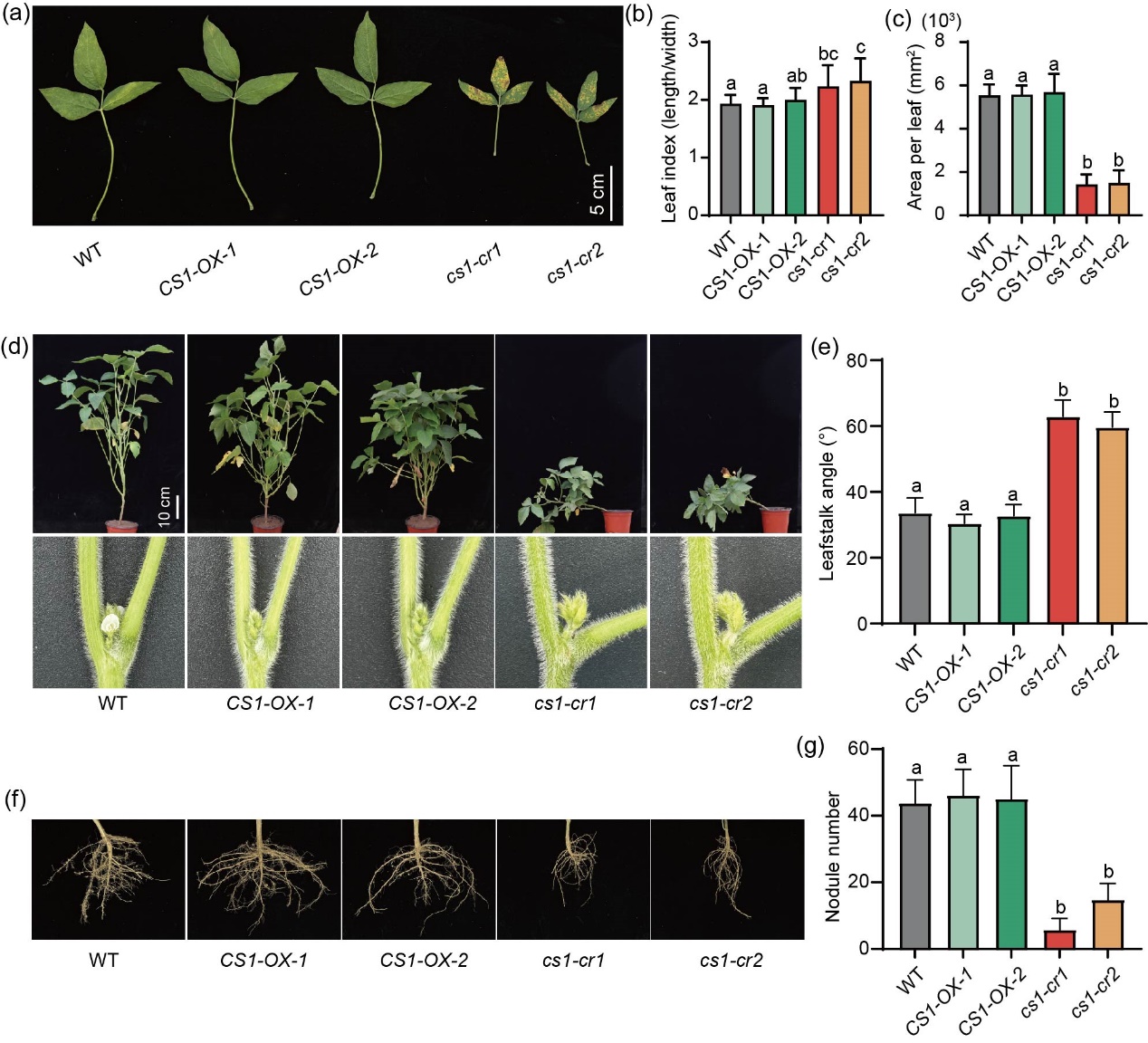
Figure S8. Effects of the *CS1* gene on soybean growth and development. (a-g) Leaf morphology (a), Leaf index (b), Area per leaf (c), Leafstalk angle (d-e) and nodule number (f-g) of the wild type (TL1), *CS1* knockout mutants (*cs1-cr1*, *cs1-cr2*), and *CS1* overexpression lines (*CS1-OX-1* and *CS1-OX-2*) at 94 days post-planting in the field. Data are presented as means ± s.d. (*n* > 9). Lowercase letters denote significant differences as determined by one-way ANOVA followed by Turkey’s post hoc test at *P* < 0.05.


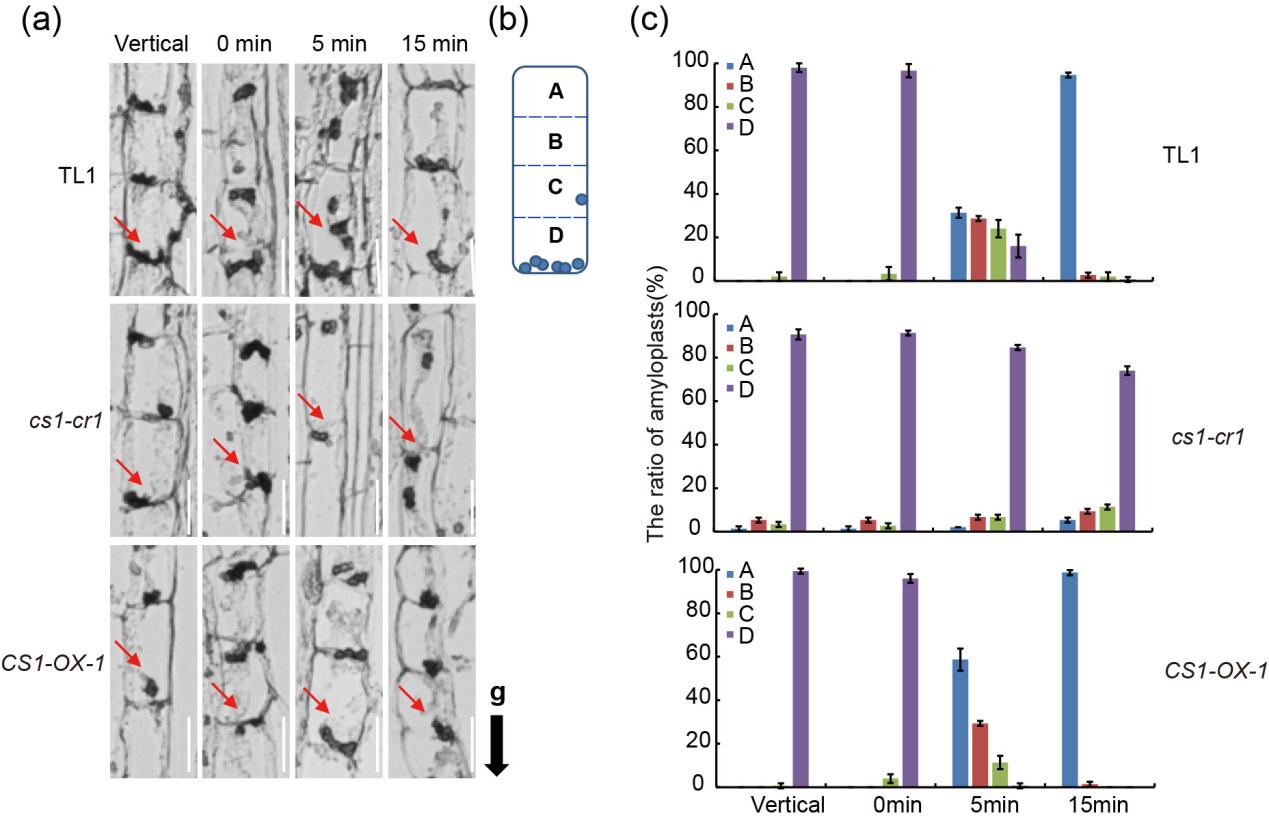
Figure S9. Amyloplast sedimentation in endodermal cells. (a)Longitudinal section images show the distribution of amyloplasts in endodermal cells (similar to Figure 1). Seven-day-old vertically growing plants were inverted for indicated durations (0 to 15 min). Hypocotyl fragments fragments (1-2 cm below the cotyledon node) were collected and fixed, with the gravity direction maintained at the indicated time point. Red arrows indicate the locations of the amyloplasts, and the black arrow indicates the direction of gravity (g). Scale bars = 5 um. (b-d) The ratio of amyloplasts in each block for TL1 (b), *cs1-cr1* (c) and *CS1-OX-1*(d). Data are means ± s.d. (*n* = 50).


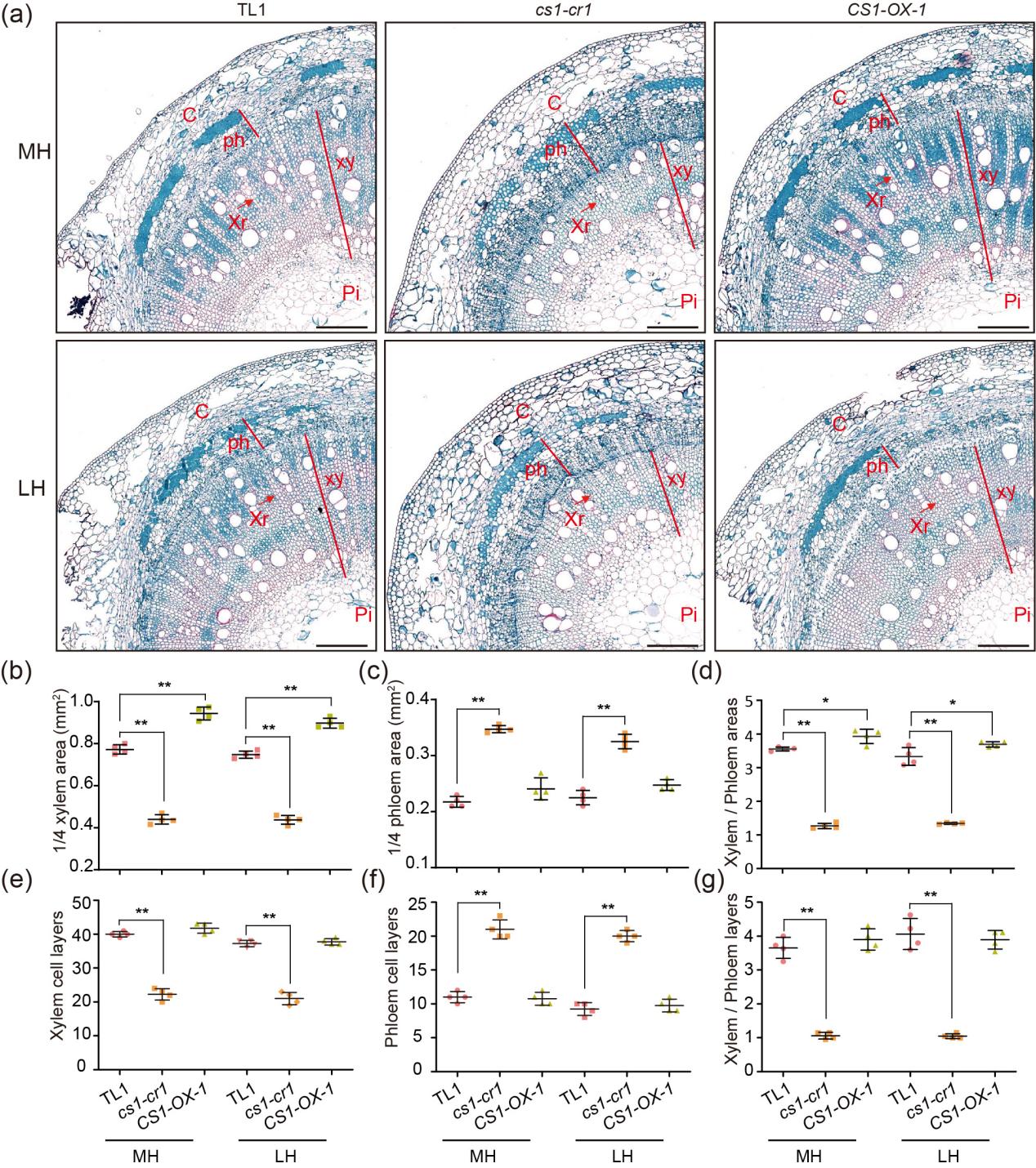
Figure S10. The *CS1* gene regulates xylem and phloem development in the hypocotyl. (a) Quadrant cross-sectional images show the cellular layer structures in the middle and lower regions of the hypocotyl. Plants of the indicated genotypes were grown under LD conditions for 7 days. C, cortex; ph, phloem; xr, xylem rays; xy, xylem; pi, pith. Red arrows indicate xylem rays. Scale bar = 250 um. (b-g) Scatter plots of xylem areas (b), phloem areas (c), xylem areas / phloem areas (d), xylem cell lays (e), phloem cell lays (f), and xylem cell lays / phloem cell lays (g) of indicated lines as in (a). Data are means ± s.d. (*n* = 4). The asterisks indicate statistically significant difference from the wild type by two-sided *t*-test (* *P* < 0.05; ** *P* < 0.01).


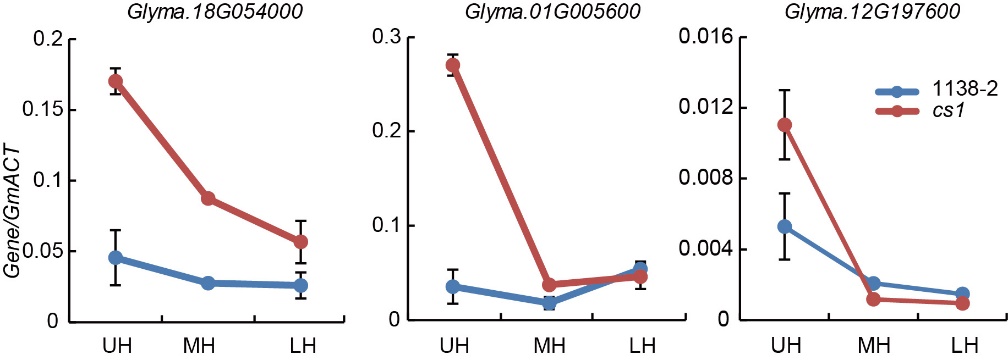
Figure S11. The expression levels of the expansin, extensin and XTH relative gene families in NN1138-2 and *cs1*. *GmActin11* was used as a control in the real-time PCR analyses. Values are means ± s.d. (*n* = 3).


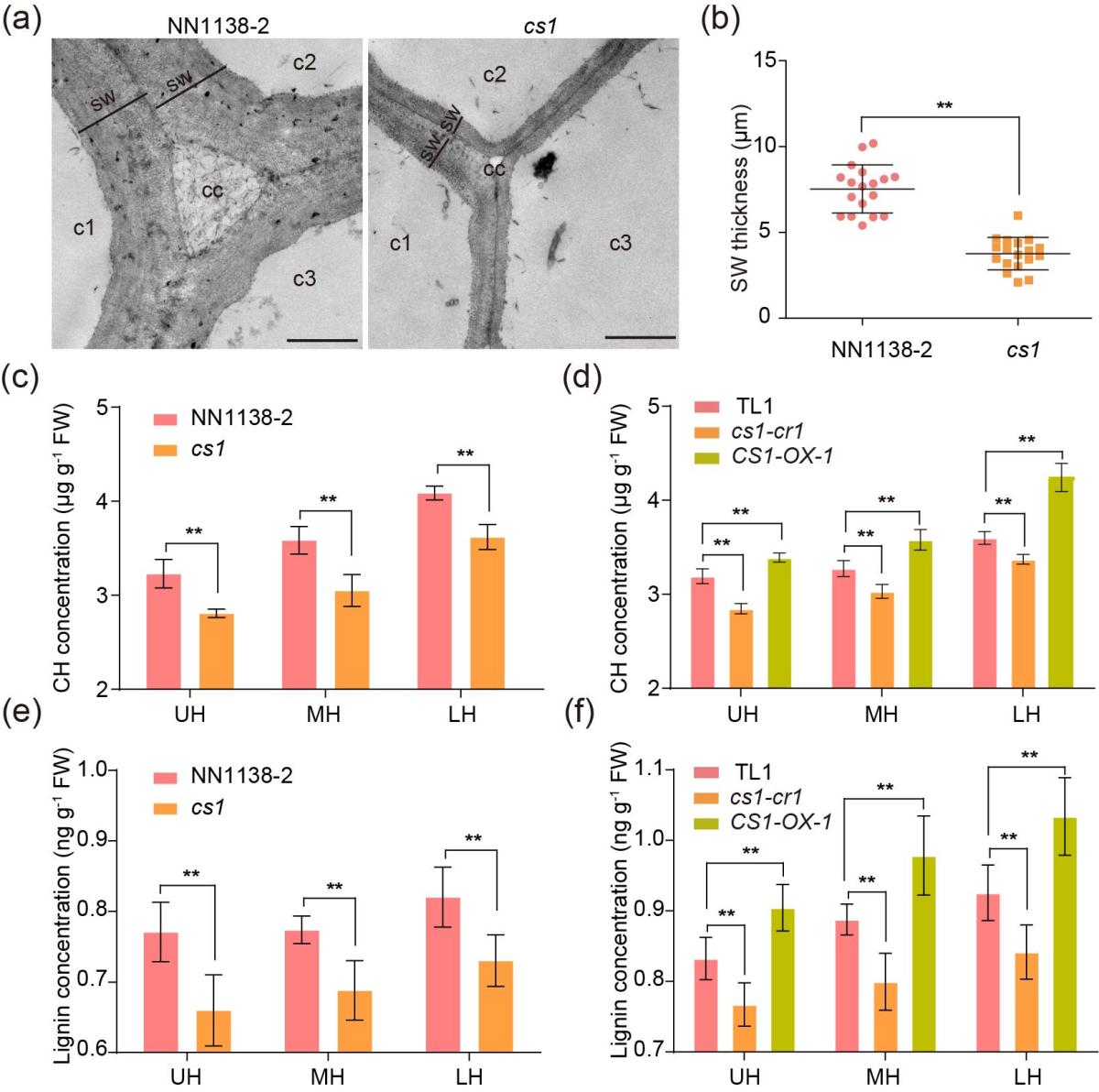
Figure S12. The *cs1* mutation leads to a reduction in endoderm secondary cell wall thickness and in the content to cellulose and lignin in the hypocotyl. (a) Transmission electron micrographs (TEM) of the upper hypocotyl endodermal cell walls from indicated NIL lines. Scale bar = 6 µm. C1~3: cell 1~3, sw: secondary cell wall, cc: cell corners, va: vacuole, ap: amyloplast. (b) The Upper hypocotyl endodermal secondary cell wall thickness (statistical results from a). Values are means ± s.d. (*n* = 18). (c-d) Cellulose and hemicellulose (CH) concentration in hypocotyl of indicated genotypes. FW means fresh weight. (e-f) Lignin concentration in hypocotyl of indicated genotypes. Values are means ± s.d. (*n* ≥ 4). The asterisks indicate statistically significant difference from the wild type by two-sided *t*-test (* *P* < 0.05; ** *P* < 0.01).


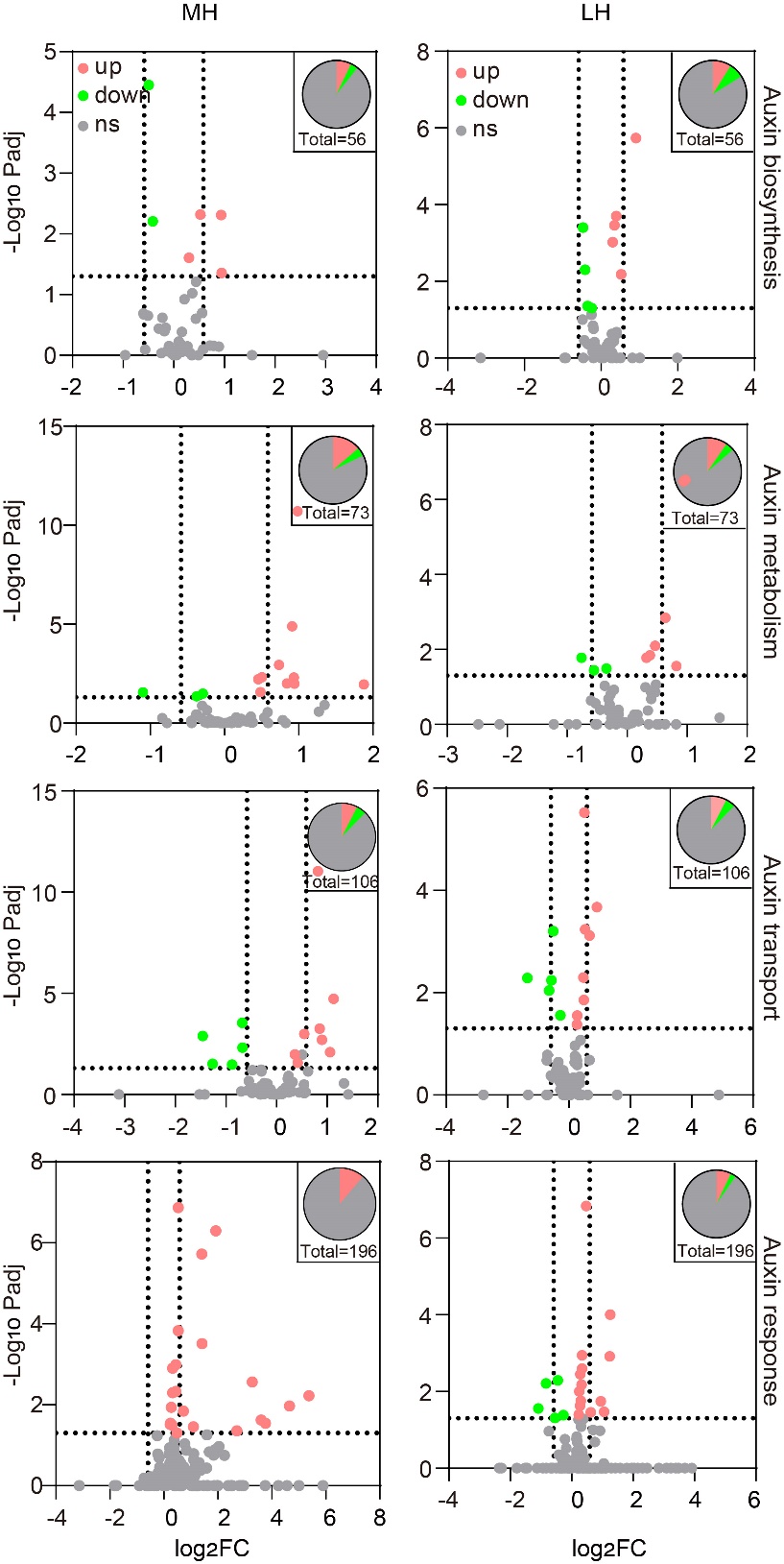


Figure S13. Volcanic plots of auxin biosynthesis, metabolism, transport and response related genes in MH and LH.


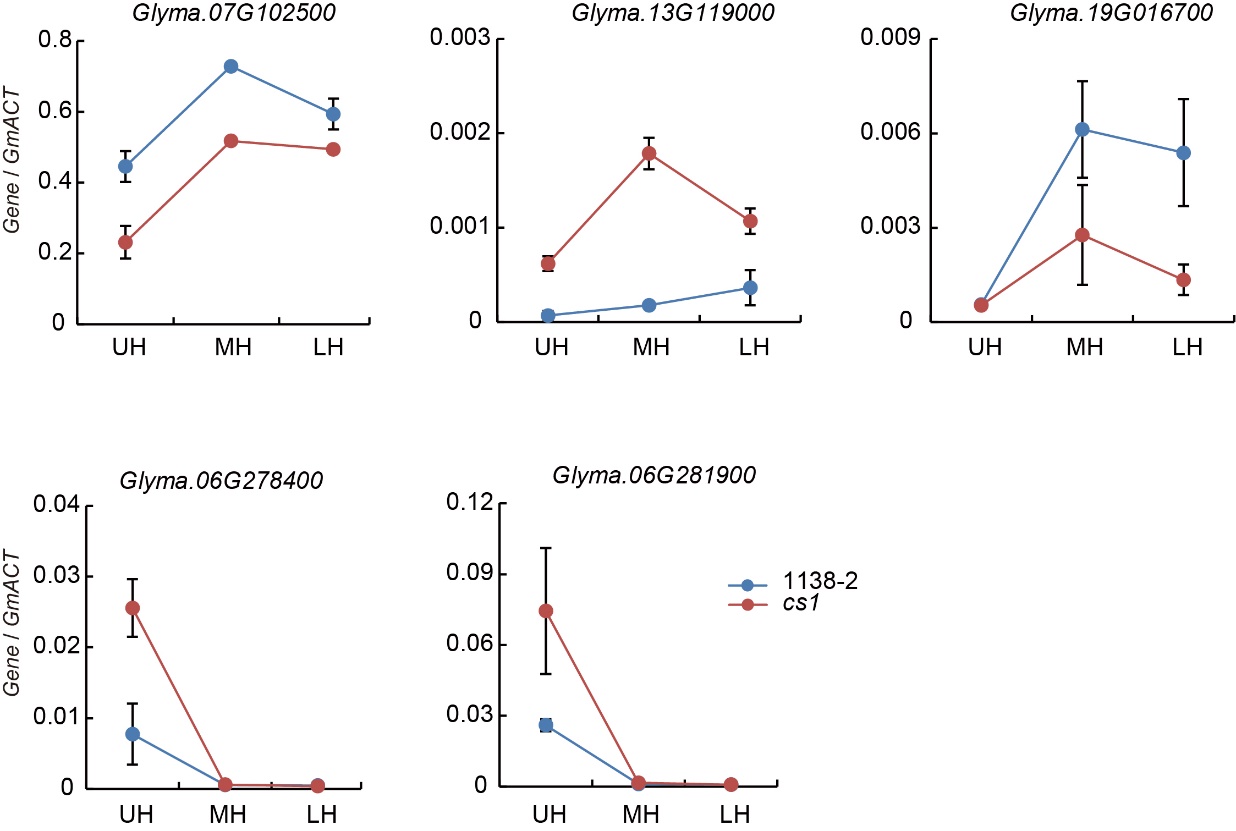
Figure S14. Expression levels of auxin transport and auxin response gene families compared between NN1138-2 and *cs1*. *GmActin11* was used as a control in the real-time PCR analyses. Values are means ± s.d. (*n* = 3).


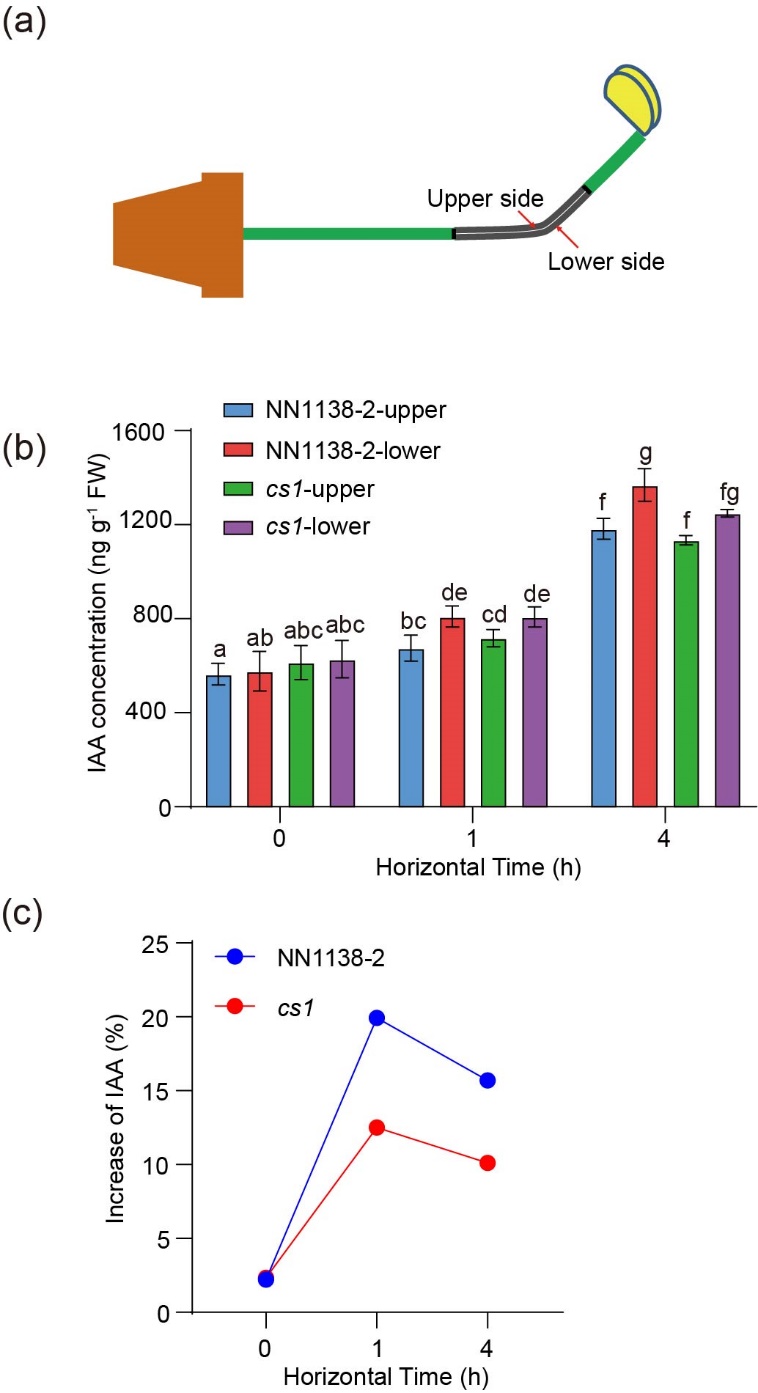


Figure S15. Asymmetric distribution of IAA concentration in hypocotyls during Gravistimulation. (a) Seven-day-old soybean seedlings were rotated 90° from the vertical to horizontal position and incubated for 0, 1, and 4 h, respectively. The gray area represents the site that was harvested at each time point. Hypocotyls were divided into their lower and upper sides along the midline (indicated by a white line). (b) IAA concentration on the upper and lower sides of NN1138-2 and *cs1* after gravistimulation. FW means fresh weight. Values are means ± s.d. (*n* ≥ 4). (c) The increase in IAA after gravistimulation is shown as percentage change, calculated as [(lower-upper)/upper]*100%.


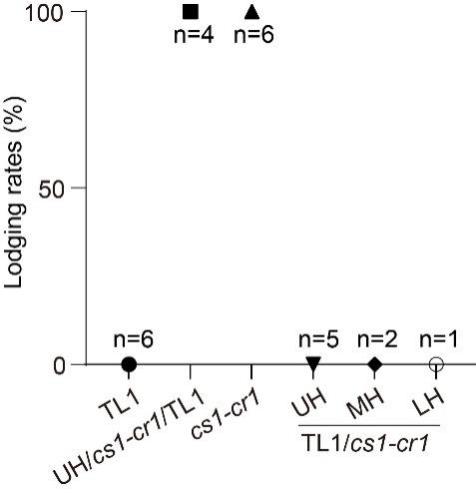
Figure S16. Lodging rate assessment of grafted plants. UH, MH and LH are the grafting sites, n is the number of grafted plants.


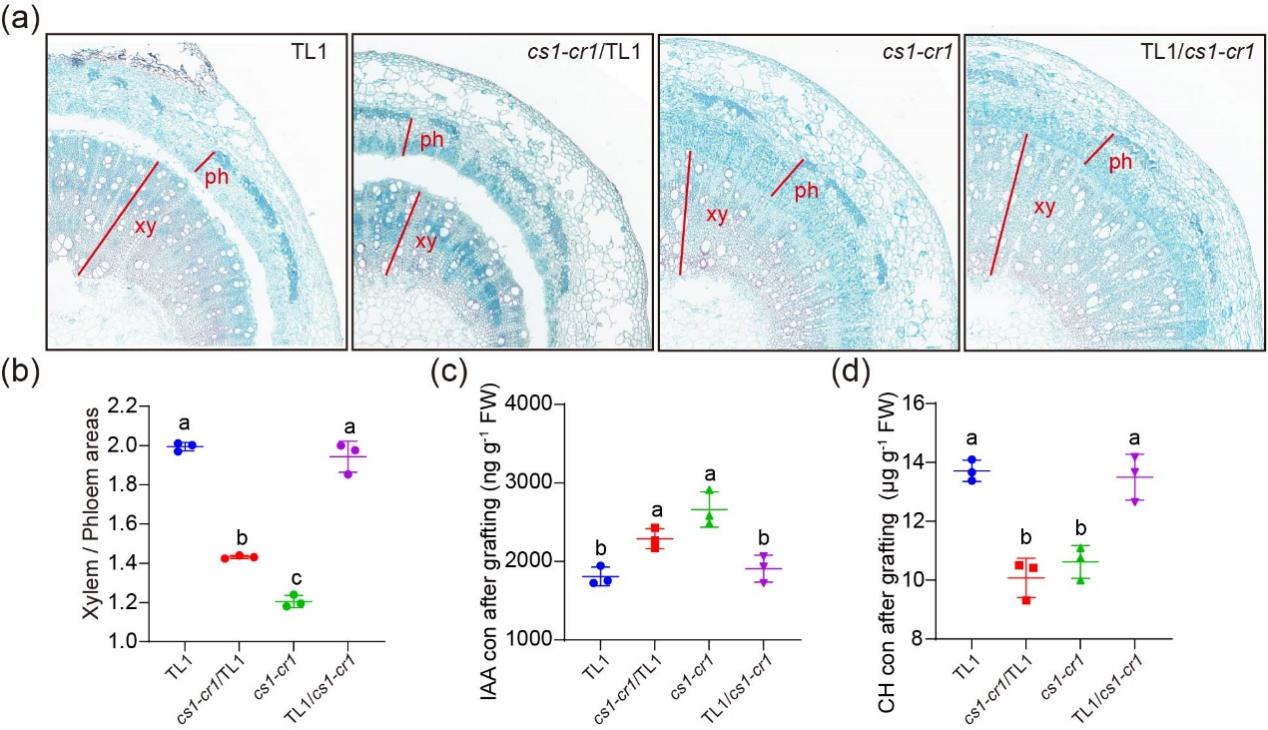
Figure S17. Structural changes and hormone determination in the rootstock following grafting. (a) Quadrant cross-sectional images show the cell layer structures within the hypocotyl rootstock. Ph, phloem; xy, xylem. (b) Scatter plots represent the ratio of xylem areas to phloem areas. (c) Auxin concentration measurements in hypocotyl rootstock of indicated lines. (d) Cellulose and hemicellulose (CH) concentration in hypocotyl rootstock of indicated lines. *cs1-cr1*/TL1: *cs1-cr1* is scion, TL1 is rootstock; TL1/ *cs1-cr1*:TL1 is scion, *cs1-cr1* is rootstock. The letters above the bars indicate significant differences (*P* < 0.05) as determined by one-way ANOVA following by Tukey's multiple comparisons test.


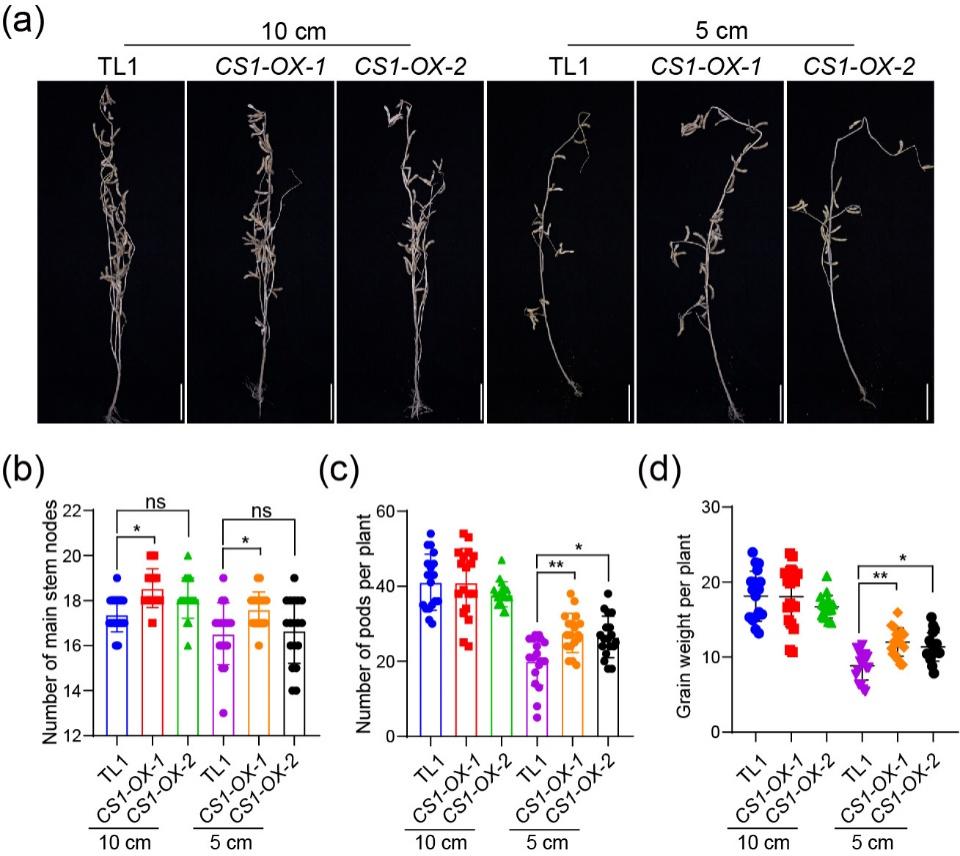


Figure S18. Phenotypic and agronomic traits of *CS1* overexpression lines under dense planting conditions in the field. (a) Field phenotype of *CS1* overexpression lines at 10 cm and 5 cm plant spacing. Scale bars, 10 cm. (b-d) Number of main stem nodes (b), number of pods per plant (c) and grain weight per plant (d) of indicate lines. Data represent means ± s.d. (*n* ≥ 15). Asterisks denote statistically significant difference from the wild type as determined by a two-sided *t*-test (* *P* < 0.05; ** *P* < 0.01).


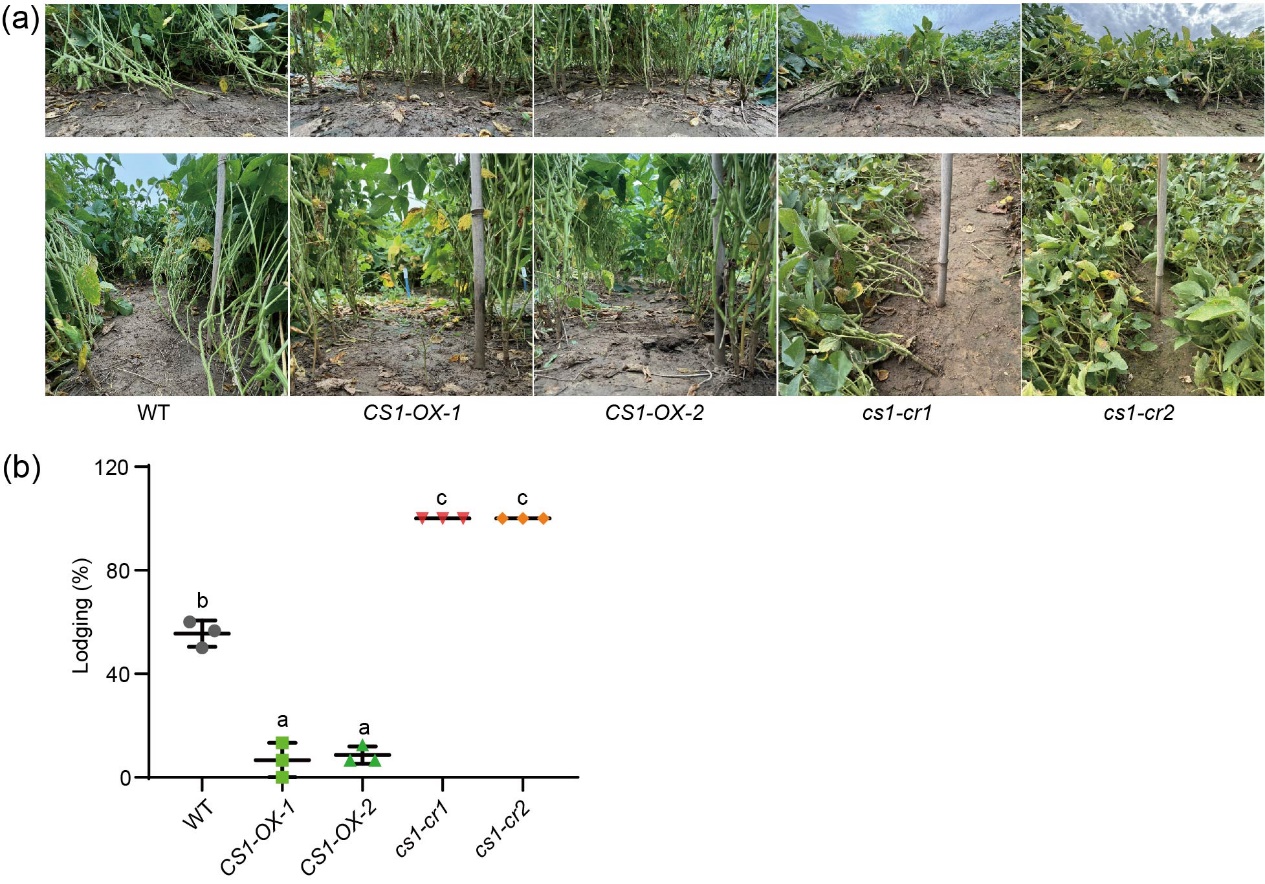
Figure S19. Lodging resistance in *CS1* overexpression lines in the field with soft oil. (a) Gross images of the indicated lines at 4 months post planting in Beijing with vertical Bamboo poles used to indicate plant inclination. (b) Lodging rate comparison of TL1, *CS1-OX lines* and *cs1-cr1* mutants at 12 cm plant interval. Plants with a main stem inclination exceeding 45° were considered lodged. Data represent means ± s.d. (*n* ≥ 15). Lowercase letters denote significant differences as determined by one-way ANOVA followed by Turkey’s post hoc test at *P* < 0.05.

.


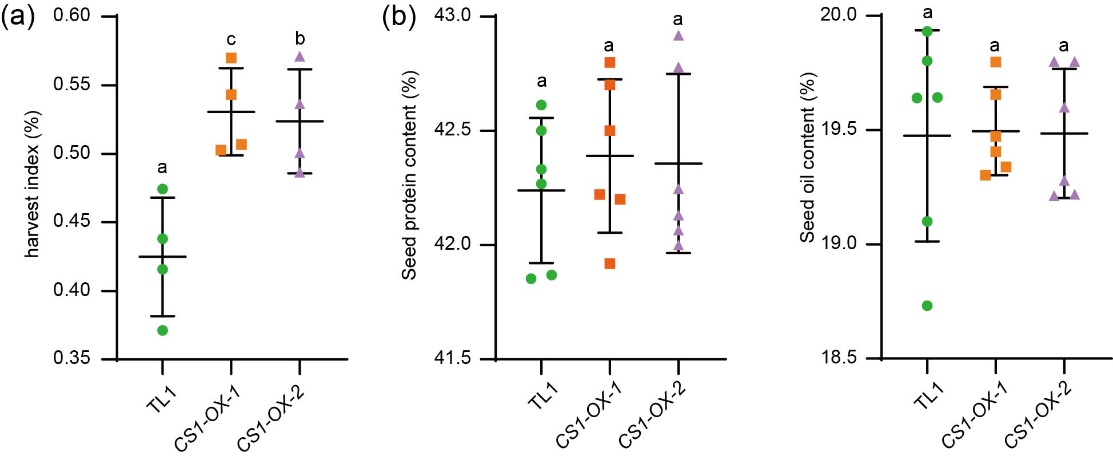


Figure S20. Evaluation of harvest index and seed quality in *CS1* overexpression lines. (a) Harvest index, which refers to the ration of grain yield to total biomass, of *CS1* overexpression lines. (b) Seed protein content and seed oil content of *CS1* overexpression lines, expressed as percentages. Data are represented as means ± s.d. (*n* ≥ 15). Lowercase letters indicate significant differences as determined by one-way ANOVA followed by Turkey’s post hoc test at *P* < 0.05.


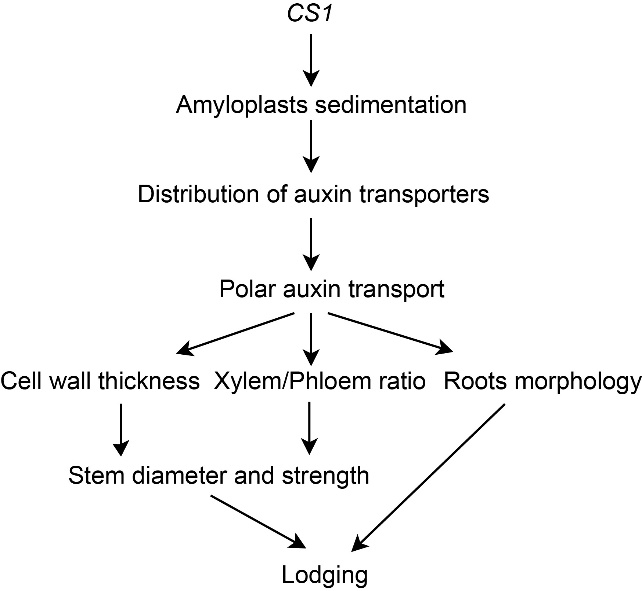
Figure S21. Schematic diagram illustrating the regulatory role of *CS1* in preventing lodging. Note: The arrows indicates the direction and correlation between the various factors influencing lodging resitance.

Table S1: Goodness-of-fit test for segregation ratios of erect to creeping trait in four crosses between the normal parents and the mutant *cs1*.

| Population | No. of WT plants | No. of MT plants | Expected ratio | χ^2^ | P |
| --- | --- | --- | --- | --- | --- |
| Williams 82 × *cs1* | 1251 | 212 | 3:1 | 85.62 | 0.00 |
| NG94-156 × *cs1* | 209 | 45 | 3:1 | 6.80 | 0.01 |
| KF1 × *cs1* | 485 | 98 | 3:1 | 20.42 | 0.00 |
| NN1138-2 × *cs1* | 548 | 116 | 3:1 | 19.68 | 0.00 |
|  | No. of segregating lines | No. of non-segregating lines | Expected ratio | χ^2^ | P |
| NN1138-2 × *cs1* F_2:3_ | 34 | 17 | 2:1 | 0.02 | 0.88 |
| KF1 × *cs1* F_2:3_ | 29 | 15 | 2:1 | 0.01 | 0.94 |

Table S2: Names and corresponding primer sequences of SSR markers for mapping *cs1* gene.

| SSR name | Shorthand | Forward primer（5'-3'） | Reverse primer（5'-3'） |
| --- | --- | --- | --- |
| BARCSOYSSR_19_1071 | S1071 | CGCACCCCTCATCCTATGTA | CCAACTAATCCCAGGGACTTACTT |
| BARCSOYSSR_19_1112 | S1112 | TCCCAAAAGCATTGAG | TATGCACGGAAGAGGA |
| BARCSOYSSR_19_1176 | S1176 | GGAGCATACAGCCTTAAGAGAT | TGGGCATCAAAACTAAGAAAA |
| BARCSOYSSR_19_1214 | S1214 | GCGGTTACATCTTGCAAACTAAATTAAC | GCGGAATTTTGCACATAAATTAATAACT |
| BARCSOYSSR_19_1230 | S1230 | GCGGACGAATTTTCCAGA | GCGGGGCAACAATATTTGAATCTA |
| BARCSOYSSR_19_1251 | S1251 | GCGAAAATGGCAGAGATAA | AATGCTAAAAGAGGAATGAAATAA |
| BARCSOYSSR_19_1254 | S1254 | CAGTGGCTTTATGGTTCTTCATC | TGGGTGTAAATAACAAGAGCCT |
| BARCSOYSSR_19_1276 | S1276 | GGGCATACGCATCCATAATC | TGCTGTTGAAGATCGGTGAG |
| BARCSOYSSR_19_1279 | S1279 | TCAACCCCTTTATTTCTTCCTTT | CGAGGACGAAAGAATATGCC |
| BARCSOYSSR_19_1285 | S1285 | TCAAAATCATTTATTACCTCATTCAT | TGAGGATATGAAATTGAATAAAGACA |
| BARCSOYSSR_19_1289 | S1289 | GGGCTAACAAGCTAAGTTGGA | AAAAAGTTTGGAGGGAGGTATT |
| BARCSOYSSR_19_1290 | S1290 | GTAGACTCTTACGAATCGAGTTTATG | GCATAAGCACTAAACGAATAGGAA |
| BARCSOYSSR_19_1302 | S1302 | TCTTTTCGATAATGCATGTCAC | GCTGTCAACTGCAGGTTCTG |
| BARCSOYSSR_19_1303 | S1303 | AAAATTAACACTAACTACCTCACGAGT | CATATTCCTCCATGCGACCT |
| BARCSOYSSR_19_1304 | S1304 | TTGGGTAGGATAATCCAATAAAAA | CCCATCTATTGAAGGGGTGA |
| BARCSOYSSR_19_1305 | S1305 | TGAATTGAAGCCAGTTGCAT | TGATAGACGTTGTCCAACAAAA |
| BARCSOYSSR_19_1308 | S1308 | TGTCGTCTTGCATTGGTCAT | ATATCCACTTCCCCCGAAAC |
| BARCSOYSSR_19_1321 | S1321 | CCATCCTTGGAACTGCTGAT | AGTTCGCATCTTTCAGGACG |
| BARCSOYSSR_19_1329 | S1329 | GCGTTGCTTGCTAAGTAGTGTTTTTAATCCT | GCGTCTCCCATCATGCAACTTCAATA |
| BARCSOYSSR_19_1427 | S1427 | TGGCAGCACACCTGCTAAGGGAATAAA | GCGAGGTGGTCTAAAATTATTACCTAT |

Table S3: Candidate genes and corresponding functions in the target mapping region of *cs1*.

| Number | Locus name | Physical location (bp) | Function annotations |
| --- | --- | --- | --- |
| ORF1 | Glyma.19G185900 | 44436187-44441423 | Carbonic anhydrase |
| ORF2 | Glyma.19G186000 | 44453524-44457822 | Metabolic process |
| ORF3 | Glyma.19G186100 | 44462011-44463438 | Transport |
| ORF4 | Glyma.19G186200 | 44483640-44485048 | Metal ion binding |
| ORF5 | Glyma.19G186300 | 44488767-44492762 | Signal transduction |
| ORF6 | Glyma.19G186400 | 44498441-44500784 | Phosphatidylinositol phosphate kinase activity |
| ORF7 | Glyma.19G186500 | 44498479-44499750 | No annotation |
| ORF8 | Glyma.19G186600 | 44503260-44512222 | Protein binding |
| ORF9 | Glyma.19G186700 | 44513435-44515497 | Plant-type cell wall organization |
| ORF10 | Glyma.19G186800 | 44518681-44523540 | PWWP domain |
| ORF11 | Glyma.19G186900 | 44527969-44564136 | Maestro-related heat domain-containing |
| ORF12 | Glyma.19G187000 | 44569958-44571763 | Transferase activity, transferring hexosyl groups |
| ORF13 | Glyma.19G187100 | 44575261-44576953 | Transferase activity, transferring hexosyl groups |
| ORF14 | Glyma.19G187200 | 44576954-44577576 | No annotation |
| ORF15 | Glyma.19G187300 | 44580708-44581301 | No annotation |
| ORF16 | Glyma.19G187400 | 44581752-44583387 | Transferase activity, transferring hexosyl groups |
| ORF17 | Glyma.19G187500 | 44585422-44587823 | Transferase activity, transferring hexosyl groups |
| ORF18 | Glyma.19G187600 | 44588115-44589512 | UDP-glucoronosyl and UDP-glucosyl transferase |
| ORF19 | Glyma.19G187700 | 44593059-44594519 | UDP-glucoronosyl and UDP-glucosyl transferase |
| ORF20 | Glyma.19G187800 | 44594914-44598298 | Pectinesterase activity |
| ORF21 | Glyma.19G187900 | 44599748-44609219 | Protein binding |
| ORF22 | Glyma.19G188000 | 44613447-44614173 | No annotation |
